# Treatment with a DC-SIGN ligand reduces macrophage polarization and diastolic dysfunction in the aging female but not male mouse hearts

**DOI:** 10.1007/s11357-020-00255-4

**Published:** 2020-08-26

**Authors:** JoAnn Trial, Rodrigo Diaz Lankenau, Aude Angelini, Jorge E. Tovar Perez, George E. Taffet, Mark L. Entman, Katarzyna A. Cieslik

**Affiliations:** 1 Department of Medicine, Cardiovascular Research, Baylor College of Medicine, One Baylor Plaza, MS: BCM 620, Houston, TX 77030 USA; 2 Texas A&M University, 2121 W. Holcombe Blvd, Houston, TX 77030 USA; 3 The DeBakey Heart Center, Houston Methodist Hospital, 6565 Fannin Street, Houston, TX 77030 USA

**Keywords:** Aging, Heart, Macrophages, DC-SIGN, Fibrosis

## Abstract

Cardiac diastolic dysfunction in aging arises from increased ventricular stiffness caused by inflammation and interstitial fibrosis. The diastolic dysfunction contributes to heart failure with preserved ejection fraction (HFpEF), which in the aging population is more common in women. This report examines its progression over 12 weeks in aging C57BL/6J mice and correlates its development with changes in macrophage polarization and collagen deposition.

Aged C57BL/6J mice were injected with dendritic cell–specific intercellular adhesion molecule-3-grabbing nonintegrin (DC-SIGN) ligand 1 (DCSL1, an anti-inflammatory agent) or saline for 12 weeks. Echo and Doppler measurements were performed before and after 4 and 12 weeks of treatment. DCSL1 prevented the worsening of diastolic dysfunction over time in females but not in males. Cardiac single cell suspensions analyzed by flow cytometry revealed changes in the inflammatory infiltrate: (1) in males, there was an increased total number of leukocytes with an increased pro-inflammatory profile compared with females and they did not respond to DCSL1; (2) by contrast, DCSL1 treatment resulted in a shift in macrophage polarization to an anti-inflammatory phenotype in females. Notably, DCSL1 preferentially targeted tumor necrosis factor-α (TNFα^+^) pro-inflammatory macrophages. The reduction in pro-inflammatory macrophage polarization was accompanied by a decrease in collagen content in the heart.

Age-associated diastolic dysfunction in mice is more severe in females and is associated with unique changes in macrophage polarization in cardiac tissue. Treatment with DCSL1 mitigates the changes in inflammation, cardiac function, and fibrosis. The characteristics of diastolic dysfunction in aging female mice mimic similar changes in aging women.

**Electronic supplementary material:**

The online version of this article (10.1007/s11357-020-00255-4) contains supplementary material, which is available to authorized users.

## Introduction

In clinical studies, heart failure with preserved ejection fraction (HFpEF) in older women is preceded by left ventricular diastolic dysfunction [42]. Over 75% of patients diagnosed with HFpEF are in fact older women. HFpEF, characterized by impaired relaxation of the myocardium and increased left ventricular stiffness, is associated with cardiac inflammation and fibrosis [4, 5, 19, 24, 32, 43, 48]. No medication trials have had an impact on outcomes, and so the increased study of aspects of this condition in animals is warranted [51].

We previously studied inflammation and fibrosis in the aging male mouse heart [12, 13, 50], and we reported chronic interstitial fibrosis caused by an elevated infiltration of monocytes that later transition into pro-fibrotic macrophages. These became collagen-producing cells (“fibrocytes,” which we termed “myeloid fibroblasts,” which correspond to cells previously known as “M2” macrophages). These macrophages, in addition to expressing collagen themselves, have pro-fibrotic effects because they stimulate increased production of collagen by resident mesenchymal fibroblasts. The dysregulated, low-level chronic cardiac inflammation by macrophages that is persistent in aging is dependent on monocyte chemoattractant protein-1 (MCP-1), which is upregulated in the aging heart [11], and on interleukin-6 (IL-6) [14]. We demonstrated before that while MCP-1 is necessary for leukocyte infiltration, IL-6 promotes monocyte-to-pro-fibrotic macrophage transition. In a previous paper [49], we examined a population of aged WT and MCP-1 knockout (MCP-1KO) mice. As we postulated, there was a striking reduction in monocyte-derived pro-fibrotic macrophages of bone marrow origin in the MCP-1KO hearts, but that did not eliminate resident pro-fibrotic macrophages. Concomitantly, there was also a marked reduction in the mesenchymal fibroblast population and its collagen synthesis in the aged male mouse [50]. Therefore, in the aging heart, the interaction between the myeloid-derived fibroblast/pro-fibrotic polarized macrophage and the mesenchymal fibroblast is essential for collagen expression and deposition and if not prevented or reduced may contribute to interstitial fibrosis and diastolic dysfunction. The current investigation arises in response to two important issues brought up by these studies: (1) the use of an MCP-1 deletion model present from birth does not mimic the normal process of aging and might well have skewed or altered aging in these mice, and (2) the studies were done only in male mice, but the burden of HFpEF is disproportionately borne by females. This paper directly addresses these two issues.

In the current study, we used a treatment to prevent interstitial fibrosis by inhibiting the maturation of myeloid cells rather than by genetic knockout of monocyte entry into the heart. We administered a ligand to dendritic cell–specific intercellular adhesion molecule-3-grabbing nonintegrin (DC-SIGN). DC-SIGN is a C-type lectin receptor present on dendritic cells, monocytes, macrophages, and neutrophils [1, 16]. The polycyclic aminothiazole derivative N-(2-{[4-(dimethylamino)phenyl]amino}-4′-methyl-4,5′-bi-1,3-thiazol-2′-yl) propanamide is a potent DC-SIGN ligand 1 (DCSL1) [8], alleviates fibrosis, reduces pro-fibrotic macrophage polarization, and increases anti-inflammatory interleukin-10 (IL-10) levels in the bleomycin-induced lung fibrosis model in young mice [16]. The advantage of our current study is that it uses an intervention during aging rather than knocking out a gene during the entire lifespan of an animal, which can have unforeseen consequences.

Heretofore, our investigations have been in male mice. In our current studies, we did side-by-side experiments using male and female aged mice per NIH recommendations and for clinical relevance. We performed functional studies before, during, and after treatment, using each animal as its own control. The timeline analysis over a 3-month period enabled us to see the progression of both the aging-related worsening of function and the response to treatment. We found that DCSL1 treatment resulted in a significant reduction in the progression of diastolic dysfunction in the female hearts but not in the male hearts. We also measured the numbers and types of cells (and their products) infiltrating the heart by flow cytometry to assess the level and type of inflammation present at the end of the experiments. In the male heart, there were greater levels of CD86^+^ pro-inflammatory macrophages (previously described as “M1” macrophages) than in the female heart, and more of the inflammatory cells were TNFα^neg^iNOS^+^ macrophages in the male heart. In the female heart (but not in the male heart), the DCSL1 treatment targeted only TNFα^+^iNOS^neg^ cells. The polarization into CD206^+^CD301^+^ pro-fibrotic macrophages was also reduced in females. Thus, DCSL1 reduced both types of polarization in females but not in males. Finally, mesenchymal cells including fibroblasts (CD45^neg^) expressed less collagen in DCSL1-treated female hearts compared with control females; again, males did not respond. This suggests that although our previous observation that pro-fibrotic macrophages influence mesenchymal cell–dependent fibrosis was correct [50], the specificity of inflammatory components affected determines the success of the treatment. In addition, these observations may provide a potential insight into the sex differences in age-associated diastolic dysfunction and the heterogeneous phenotypes of HFpEF.

## Methods

### Animals

Aged 18-month-old female and male C57BL/6J mice were obtained from the National Institute on Aging, and some were aged until they were 21 months old. Young 3-month-old animals were obtained from the Jackson Laboratory. The 18–21-month-old mice were injected intraperitoneally (0.01 mg/kg body weight, three times a week) with DC-SIGN ligand 1 (DCSL1) or saline for 12 weeks. DCSL1 is the polycyclic aminothiazole derivative N-(2-{[4-(dimethylamino)phenyl]amino}-4′-methyl-4,5′-bi-1,3-thiazol-2′-yl) propanamide and was obtained from ChemBridge (San Diego, CA). All animals were treated in accordance with the NIH Guide for the Care and Use of Laboratory Animals (DHHS publication (NIH) 85-23, revised 1996) and approved by the Baylor College of Medicine Institutional Animal Care and Use Committee.

### Heart function

Noninvasive measurements were performed on anesthetized mice under 1.5% isoflurane gas in oxygen on a heated ECG board. Two-dimensional (2D) and M-mode images to assess left atrial and ventricular anatomy and function were acquired using a Vevo 770 system (VisualSonics, Toronto, Canada) as we previously described [25, 34]. Mitral inflow and aortic outflow Doppler measurements were recorded using a 10-MHz pulsed probe and Doppler signal processing workstation (DSPW; Indus Instruments, Houston, TX, USA).

### Single cell preparation

Twenty-four hours after the last DCSL1 injection, animals were euthanized and hearts were excised. To preserve cell viability, only three hearts were harvested in the morning to avoid changes related to the circadian rhythm. The whole heart minus a small piece of the ventricles (about 20 mg) that was taken for histology or hydroxyproline assay was used to prepare a single cell suspension. Hearts were cut into 1-mm^3^ pieces and placed into a digestion buffer consisting of 2 mg/ml of collagenase type 4 (Worthington, #LS004188) and 2 mg/ml of dispase II (Sigma, #D4693) in DPBS with calcium and magnesium (Thermo Fisher Scientific). Heart tissue was incubated in a 37 °C shaking water bath with trituration until a single cell suspension was obtained. Cells were filtered through a 40-μm strainer into a quenching buffer containing 2% FBS (Atlanta Biologicals, #S11550) in DPBS without calcium and magnesium (Thermo Fisher Scientific). During this digestion, the myocytes disintegrate and the debris are subsequently removed by the passage of the preparation through a fine mesh, so the remaining cells are de facto a nonmyocyte population.

### Flow cytometry

After isolation, cardiac cells were washed, erythrocytes were removed using the RBC lysis buffer (BioLegend, #420301), and then the remaining cells were centrifuged. Next, cells were divided into two groups that were used to detect (1) monocytes and (2) macrophages and lymphocytes. For monocytes, cells were immediately stained with Zombie Yellow (BioLegend, #423104) to discriminate between the dead and live cells following blocking with the Fc receptor (using the anti-CD16/CD32 antibody), then proceeding to specific marker staining.

For macrophage and lymphocyte staining, cells were incubated in the RPMI 1640 medium (Invitrogen, #72400-047) supplemented with 20% FBS in the presence of brefeldin (Thermo Fisher Scientific, #00-4506-51), monensin (Sigma, #M5273), and activation cocktail (BioLegend, #423302). After 5 h, cells were collected, centrifuged, washed, and stained with Zombie Yellow, followed by appropriate external and internal marker staining. For fixation and permeabilization, we used the Foxp3/Transcription Factor Staining Buffer Set (eBioscience, #005523-00). Details regarding the antibodies used are summarized in Supplemental Table 1. Total cell events (nonmyocytes) were screened for singlet cells and viability before being analyzed for antibody positivity. Gates for positive/negative discrimination and color compensation were set using fluorescence minus one for each antibody. For some antibodies, the amount of fluorochrome-associated antibody bound to the cell was expressed as mean fluorescence intensity (MFI). Cytometry was performed on a CytoFLEX 13-color instrument (Beckman Coulter) using the associated software for analysis.

### Hydroxyproline assay

The collagen content in the heart was measured by determining the content of hydroxyproline [15]. The experiment was performed by using a colorimetric assay kit (MilliporeSigma, #MAK008), following the manufacturer’s recommendation with minor changes. Briefly, after homogenization, cardiac samples (about 20 mg of both ventricles) were weighed and resuspended in a volume of 50 μl H_2_O per 10 mg of tissue. Acid hydrolysis was then done in 6 N HCl (vol/vol) for 180 min at 120 °C. The hydrolysate was cooled and centrifuged for 3 min at 10,000×*g*. The supernatant was collected, and 25 μl was added to a 96-well plate for the assay (in duplicate for each sample).

The drying out process was then performed in an oven at 60 °C overnight. The next day, hydroxyproline was first oxidized with 7% Chloramine T in the oxidation buffer; then, Ehrlich’s solution was added in the wells (1.4 M 4-dimethylaminobenzaldehyde in 20% perchloric acid and 67% isopropanol) and the plate was incubated at 60 °C for 90 min. After cooling, absorbance was read at 560 nm. The hydroxyproline content was calculated using a standard curve of high-purity hydroxyproline and finally reported per milligram of the heart weight.

### Statistical analysis

**Fig. 1:**
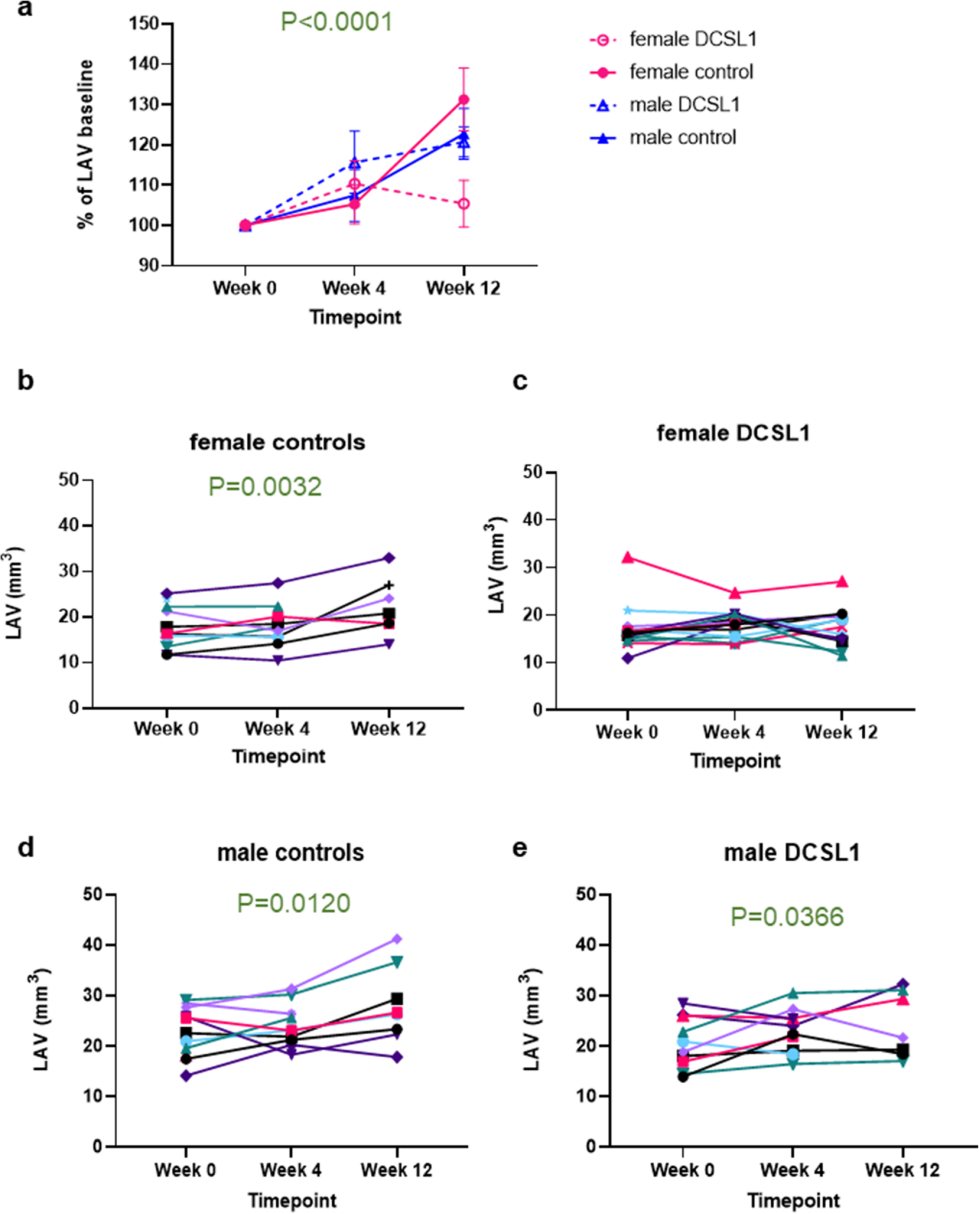
Left atrial volume (LAV) in mice subjected to 12 weeks of saline (controls) or DCSL1 injections. **a** Changes in LAV presented as a percent of baseline measurements obtained at time 0. **b**, **c** LAV in females. **d**, **e** LAV in males. **b**–**e** Graphs depict serial measurements in individual mice. Each animal serves as its own control. For graph **a**, results were presented as mean ± SEM using two-way ANOVA analysis with Geisser-Greenhouse correction, *N* = 7–9. For **b**–**e**, results are expressed as individual measurements. Statistical analysis was performed using an ordinary two-way ANOVA. Some animals died before the study was completed, but even a few time points were included in the graphs, *N* = 9–10

Data are presented as mean ± standard error of the mean (SEM). For the comparison of two groups, Student’s *t* test was performed, followed by the Mann-Whitney test if the distribution was not normal. For more than two groups, a one-way ANOVA was applied with Kruskal-Wallis correction. Data in Fig. 1 were analyzed using two-way ANOVA with Geisser-Greenhouse correction. Nonparametric tests were used when normal distribution was not present. Analyses were performed using GraphPad Prism version 8.0.

## Results

### Cardiac structure and function with aging

Our previous work [34] focused on aging of male C57BL/6J mice and extensive analyses of systolic and diastolic dysfunction with age. In the current study, which now includes females, we found that with aging from 3 to 21 months, body weight increased by 30% in the females (Supplemental Table 2a, b). Despite that increase, cardiac output, left ventricular (LV) end diastolic volume, and stroke volume were unchanged with age. LV wall thickness, both anteriorly and posteriorly, increased as did the calculated LV mass, and the increase in LV wall thickness was greater than that seen in males [34]. Fractional shortening was slightly higher in the 21-month-old females compared with young animals. The left atrial volume (LAV) increased by 30% in both the mediolateral and anterior-posterior dimensions (Supplemental Table 2a). By Doppler, the *E* peak velocity was lower in the old females and the relaxation time was longer, suggesting overall worse diastolic function (Supplemental Table 2b). The systolic parameters, peak aortic velocity, and mean velocity were decreased with age as well. The overall interpretation is that aging negatively impacted cardiac structure and function in the females.

### Cardiac structure and function of aged males and females are different

At baseline (21 months), there were significant anatomic and functional differences between males and females (see Table 1 and Supplemental Table 3a, b). Males had 19% heavier hearts, had larger aortas, and had 22% larger LAV. The male mice were 13% heavier than the females, so a component of these differences may be attributed to the differences in body mass. There were no differences in LV fractional shortening, but the preejection time, peak aortic flow velocity, and mean acceleration, which are additional measures of systolic function, were better in the males than the females. Diastolic function, as measured by the *E* peak velocity, isovolumic relaxation time (IVRT), and IVRT/RR (corrected for heart rate), was more impaired in the old females. The noninvasive assessment was that both the systolic and diastolic functions were more impaired in the old females than the males at baseline.

**Table 1:** Echo and Doppler measurements in 21-month-old females and males

	Females	Males	*t* test
Heart rate (bpm)	465 ± 8	437 ± 12	ns
Ejection fraction (%)	58.5 ± 1.2	55.9 ± 1.6	ns
LV mass (AW) corrected (mg)	126 ± 5.3	148 ± 5.6	0.00867
Preejection time (ms)	18.5 ± 0.5	16.8 ± 0.5	0.02703
Peak aortic velocity (mm/s)	755 ± 20	933 ± 27	< 0.00001
Mean acceleration (mm/s^2^)	60,203 ± 2368	70,547 ± 2818	0.00811
Aorta (mm)	1.54 ± 0.01	1.66 ± 0.02	0.00001
Body weight (g)	30.6 ± 0.8	36 ± 0.9	0.00006
LAV (mm^3^)	18.1 ± 1.0	21.7 ± 1.1	0.01856
*E* peak velocity (mm/s)	634 ± 18	624 ± 15	ns
*E* stroke distance (mm)	10.6 ± 0.4	12.0 ± 0.4	0.02215
*A* peak velocity (mm/s)	493 ± 14	452 ± 24	ns
*E*-*A* peak velocity ratio	1.29 ± 0.02	1.48 ± 0.1	ns
Isovolumic relaxation time (ms)	21.4 ± 0.7	18.4 ± 0.5	0.00169
IVRT/RR	0.16 ± 0.003	0.13 ± 0.004	< 0.00001

Abbreviations: *LV mass (AW)* left ventricle mass (calculated from anterior wall), *LAV* left atrial volume, *E peak velocity* early-wave peak velocity, *E stroke distance* early-wave stroke distance, *A peak velocity* atrial-wave peak velocity, *E-A peak velocity ratio* early-wave/atrial-wave peak velocity ratio, *IVRT/RR* isovolumic relaxation time/RR interval ratio, *ns* not statistically significant

### DCSL1 treatment preserved cardiac function in the aged females

We previously determined that the increased fibrosis in the aged male heart has a direct link to diastolic dysfunction [50]. Since it has been demonstrated that DCSL1 alleviates fibrosis and increases IL-10 levels in the bleomycin-induced lung fibrosis model in young mice [16], we tested the hypothesis that it could have the same efficacy in the aging heart. Therefore, we subjected 18–21-month-old animals to 12 weeks of DCSL1 treatment (0.01 mg/kg body weight). Twelve weeks was chosen because it allows sufficient further aging for changes in heart function to be demonstrable by our techniques. We used noninvasive methods to assess heart function so that each animal could be measured repeatedly over the 12 weeks of treatment.

We have previously demonstrated the value of LAV as a robust measure of diastolic function, reflecting time-averaged left ventricular filling pressures [34]. Because LAV is monotonic (increasing, never decreasing), we were able to look at it serially at each time point (week 0, 4, and 12) for each mouse. Expressed as percent of baseline, the LAV increased in three of the four groups by more than 25%; there was no change in the DCSL1-treated females (Fig. 1a, Supplemental Table 4a). The data for the individual mice confirm that the LAV, as a reflection of LV filling pressure, increased in both the control groups and the treated male mice (Fig. 1d, e), but the LAV remained essentially unchanged with aging in the DCSL1-treated females (Fig. 1b, c) with a degree of heterogeneity expected for older animals.

This lack of increase in LAV suggests preservation (or improvement) in diastolic filling in the DCSL1-treated female mice. By contrast, no effect of treatment on LAV was seen in the males despite the inhibition of LV hypertrophy (Fig. 1a, d, e). While LAV increased in both male groups, *E* peak velocity increased (Supplemental Table 4b) and IVRT increased slightly with DCSL1 treatment and decreased slightly over the 3 months in the control males. Rather than reflecting improved diastolic function, the changes in *E* peak velocity and IVRT may be an example of “pseudonormalization.” That is, in the context of enlarged LAV, increases in *E* peak velocity and shortening of IVRT reflect increases in left atrial pressure and worsened diastolic function [37]. Other aspects of cardiac function were unchanged, emphasizing the utility of LAV for serial assessment and interpretation of diastolic function.

### Phenotypic differences in leukocyte populations in the aged female and male mouse hearts

**Fig. 2:**
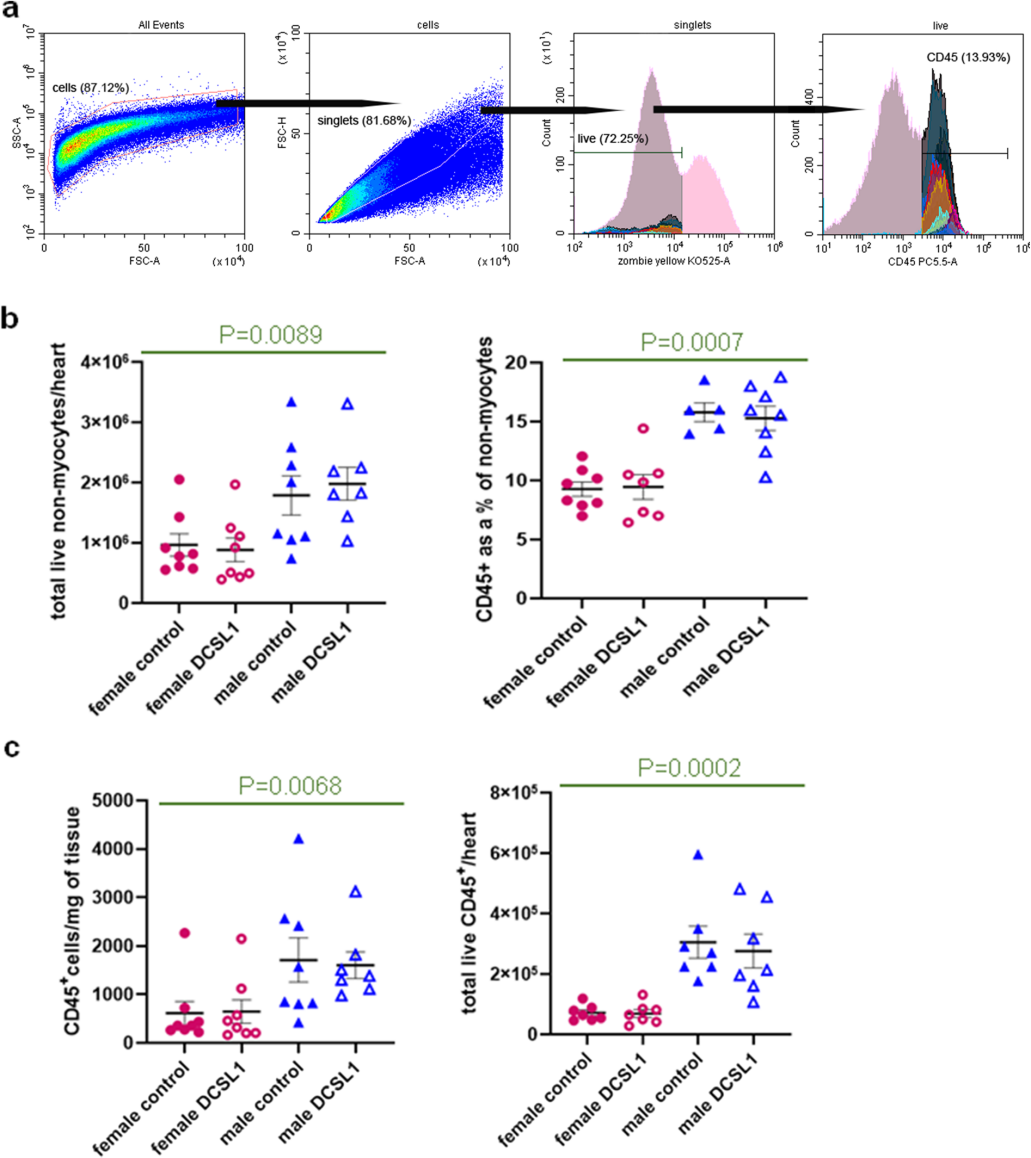
Differences in the total number of nonmyocytes and CD45^+^ cells in the aged female and male hearts quantified by flow cytometry. **a** Gating strategy. Debris (SSC-A versus FSC-A) and doublets (FSC-H versus FSC-A) were excluded; then, live/dead discrimination was determined using Zombie Yellow. CD45^+^ cells were then further subgated on CD11b^+^ cells (for monocytes only). **b** The total number of live nonmyocytes per heart (left panel) and CD45^+^ cells as a percent of live nonmyocytes (right panel). **c** The total number of CD45^+^ leukocytes per milligram of tissue (left panel) and the total number of live CD45^+^ cells per heart (right panel). *N* = 8–5 per each group. Results are expressed as mean ± SEM. Statistical analysis was performed using two-way ANOVA with Kruskal-Wallis correction

As we report in Table 1, cardiac function exhibits age-related [34] and sex-specific differences. We had reported that a link between inflammation and cardiac fibrosis has been described before in male mice [50]. In this study, we examined a more detailed assessment of the inflammatory response in the aging male and female mice. Using flow cytometry (the gating strategy is detailed in Fig. 2a), we investigated the leukocytes in the aging male and female hearts after confirming that the numbers of white cells in the blood were no different in the two sexes (Supplemental Fig. 1).

### Dendritic cell assessment

One of the targets of DCSL1 may be the dendritic cell, which, along with other myeloid cells, expresses DC-SIGN. To identify dendritic cells, we analyzed the heart cells for those expressing CD45 and CD11b in combination with either CD103 or CD11c or both. Female hearts contained a higher number of CD103^+^ cells and a smaller increase of CD11c^+^ and double-positive cells over that in males. Neither of these numbers was affected at all by DCSL1 treatment (Supplemental Table 5).

### Lymphocyte assessment

To increase the confidence that the dendritic cells were not the target of the DCSL1, we analyzed the T lymphocyte and NK cell numbers, which might have been affected by any change in the status of the dendritic cells in their antigen presentation capacity. The major differences in T cells and their subsets were seen when comparing males and females. There was an increase in the total numbers of CD3^+^ T cells in males, and this was the only measure affected by DCSL1 treatment (Supplemental Table 6). Males had fewer numbers of CD3^+^CD4^+^ cells but higher numbers of CD56^+^ NK cells and CD3^+^CD8^+^ T cells. There were also much higher numbers of CD3^+^CD8^+^ cells bearing CXCR3 in males. Most of the numbers were not altered by DCSL1 treatment (Supplemental Table 6).

### Differences in the cardiac monocyte subpopulations between males and females

The enzymatic digestion of the heart to yield viable cells destroys the myocytes, so we call the resulting preparation of cells “nonmyocytes.” The numbers of nonmyocytes and leukocytes (CD45^+^) in the aging female and male hearts were different (Fig. 2b, c and Supplemental Fig. 3a), with the males exhibiting higher numbers of total nonmyocytes (Fig. 2b (left panel)) and leukocytes (Fig. 2b (right panel) and c), consistent with the larger heart size in the males (Table 1). However, DCSL1 treatment did not alter the total number of cells in these two populations (Fig. 2b–d).

**Fig. 3:**
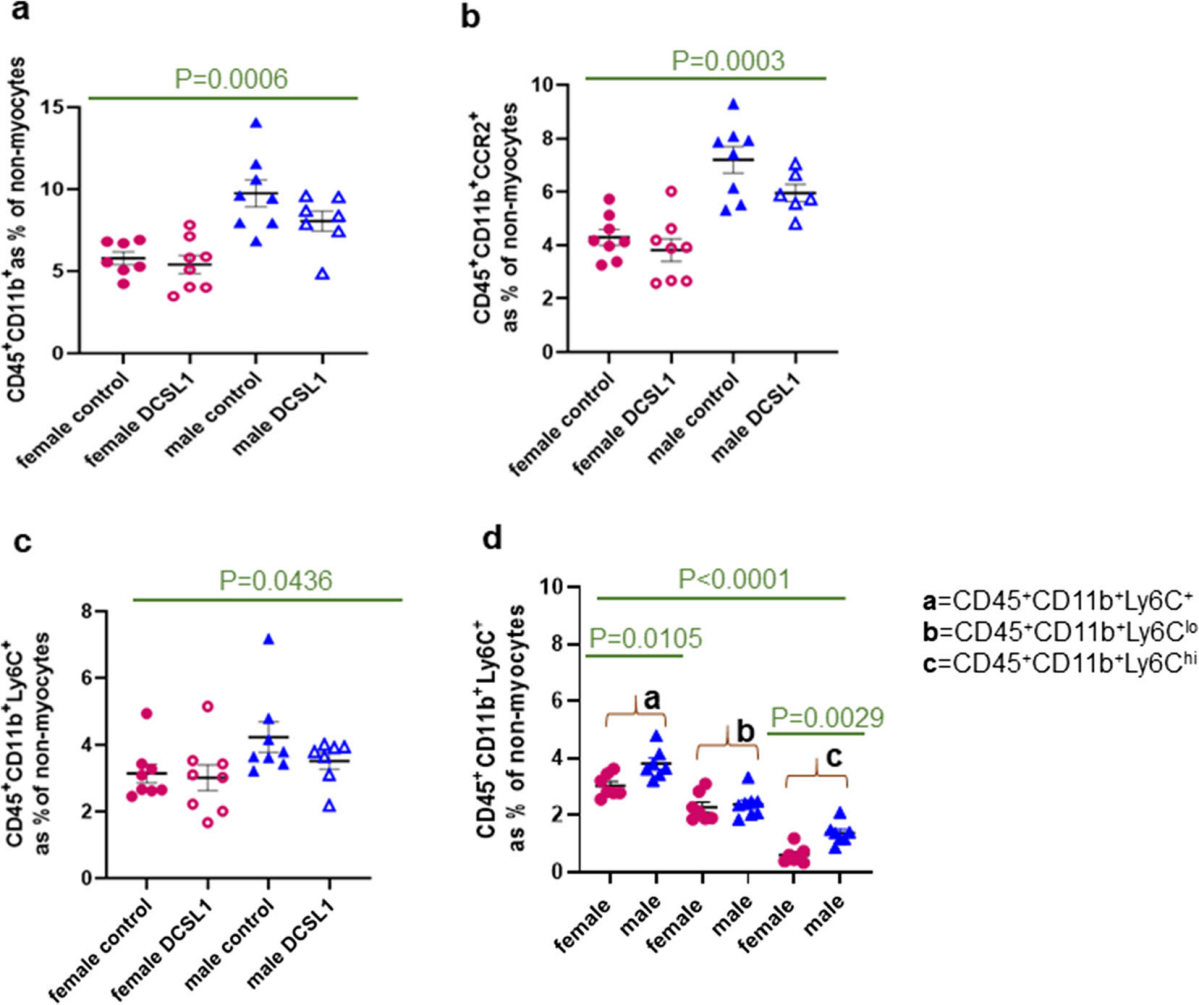
Differences in the cardiac myeloid population in the aged female and male hearts quantified by flow cytometry. **a** CD45^+^CD11b^+^ cell quantification. **b** CD45^+^CD11b^+^CCR2^+^ monocyte quantification. **c** CD45^+^CD11b^+^Ly6C^+^ cell quantification. **d** Ly6C and its frequency of the total and low and high subtypes in the cardiac leukocyte population. Results are expressed as mean ± SEM. Statistical analysis was performed using Student’s *t* test followed by the Mann-Whitney test. *N* = 7–8 animals per group

The monocyte subpopulations in the heart (Fig. 3) were different in males and females. The aging male heart has a higher number of CD45^+^CD11b^+^, CD45^+^CD11b^+^CCR2^+^, and CD45^+^CD11b^+^Ly6C^+^ cells, and these populations were not affected by DCSL1 treatment (Fig. 3a–c). We also found that the aging male myocardium contained almost a threefold higher number of Ly6C^hi^ monocytes than the female myocardium (Fig. 3d). The ratio of Ly6C low to high in males was 1.44, whereas the same ratio in females was 2.77, indicating greater entry of pro-inflammatory cells into the male heart. Ly6C^hi^ monocytes employ CCR2 to enter the site of inflammation [45], and in the male heart, we found an increased number of cells expressing CCR2 (Fig. 3b). Because the number of CCR2^+^ cells was higher in the aged male hearts, it may indicate increased monocyte infiltration in response to MCP-1. We previously reported elevated expression of MCP-1 in the aging male heart [11]. From these results, we concluded that the environment of the aging male heart is more inflammatory than that of the female heart. No significant change was found for DCSL1-treated mice, neither in the male or female group (Fig. 3). From these results, we concluded that DCSL1 did not act to change the monocyte phenotype. Considering these findings, the next step was to investigate the various macrophage populations that may transition from monocytes, especially since this aspect of myeloid biology was described previously as a target for DCSL1 [16].

### DCSL1 reduces both pro-inflammatory and pro-fibrotic macrophage polarization in females

**Fig. 4:**
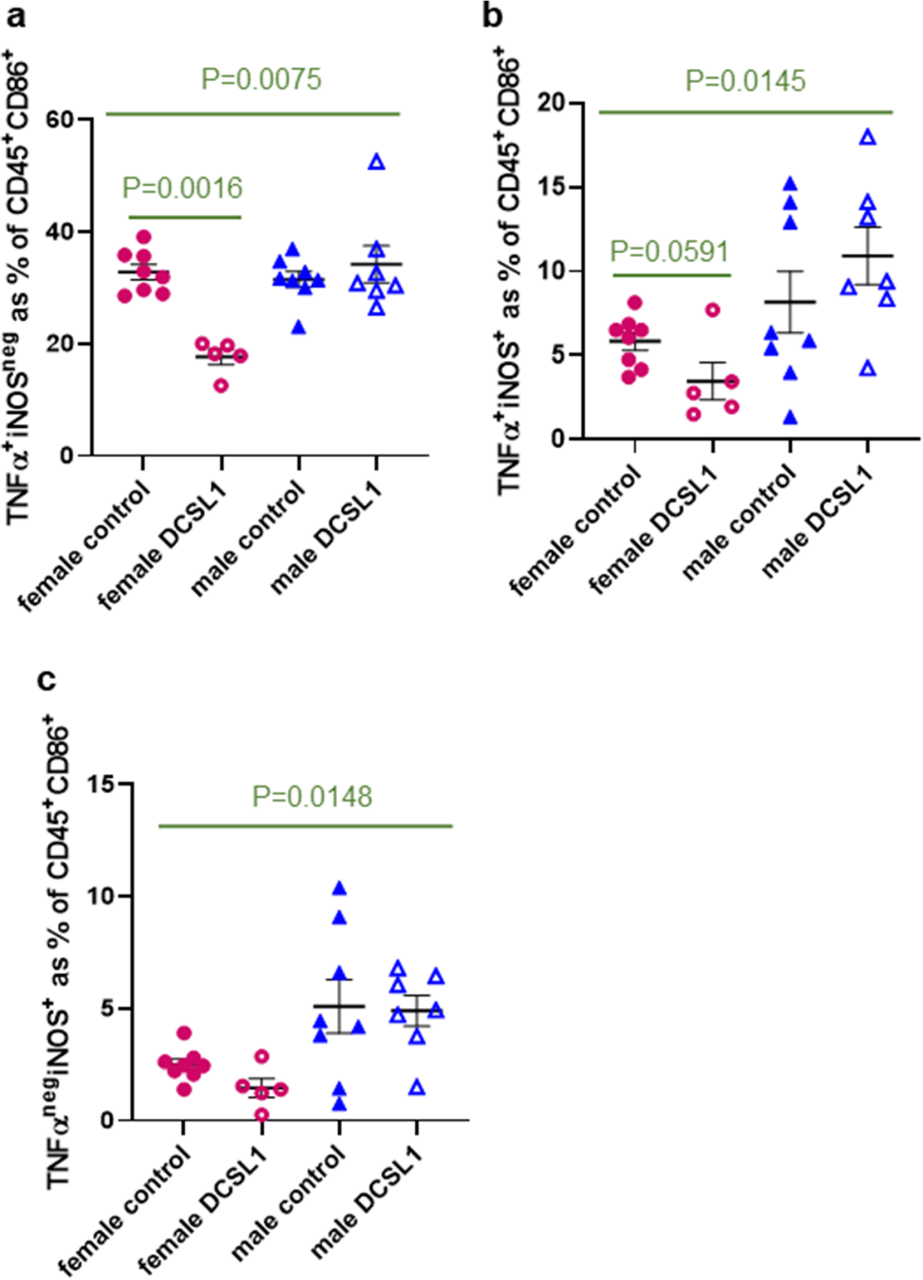
DCSL1 treatment reduces inflammatory macrophage polarization by specifically targeting TNFα^+^ but not iNOS^+^ cells in female hearts. Quantification of the **a** TNFα^+^iNOS^neg^, **b** TNFα^+^iNOS^+^, and **c** TNFα^neg^iNOS^+^ populations as a percent of CD45^+^CD86^+^ leukocytes. For more than two groups, a two-way ANOVA was applied with Kruskal-Wallis correction. For only two groups (to compare controls with DCSL1-treated mice), we used Student’s *t* test followed by the Mann-Whitney test as statistical analysis. Results are presented as mean ± SEM. *N* = 5–8 for females and *N* = 7 for males

In young animals, pro-inflammatory macrophages usually are characterized by not only CD86 expression but also tumor necrosis factor-alpha (TNFα) and inducible nitric oxide synthase (iNOS). By contrast, in our aging animals, there was dissociation in the CD86^+^ population, with some of these cells being positive for one only or both of the markers (Fig. 4). Surprisingly, there was an increase in total iNOS^+^ cells in control males versus females (14.0% versus 8.3%), whereas the total percent of TNFα^+^ cells in untreated mice of both sexes was similar (~40% of CD45^+^CD86^+^ cells). We also found that there was a marked decrease in the number of unique pro-inflammatory macrophages defined as TNFα^+^iNOS^neg^ with DCSL1 treatment in females but not in males (Fig. 4a). The number of TNFα^+^iNOS^+^ cells in DCSL1-treated female hearts was also somewhat reduced (not statistically significant, *P* = 0.06) but not in males (Fig. 4b). Immunofluorescence staining of CD45^+^TNFα^+^ cells in a heart section of the control and DCSL1-treated female hearts is shown in Supplemental Fig. 3b. DCSL1 treatment had no effect on the TNFα^neg^iNOS^+^ population in either sex (Fig. 4c).

**Fig. 5:**
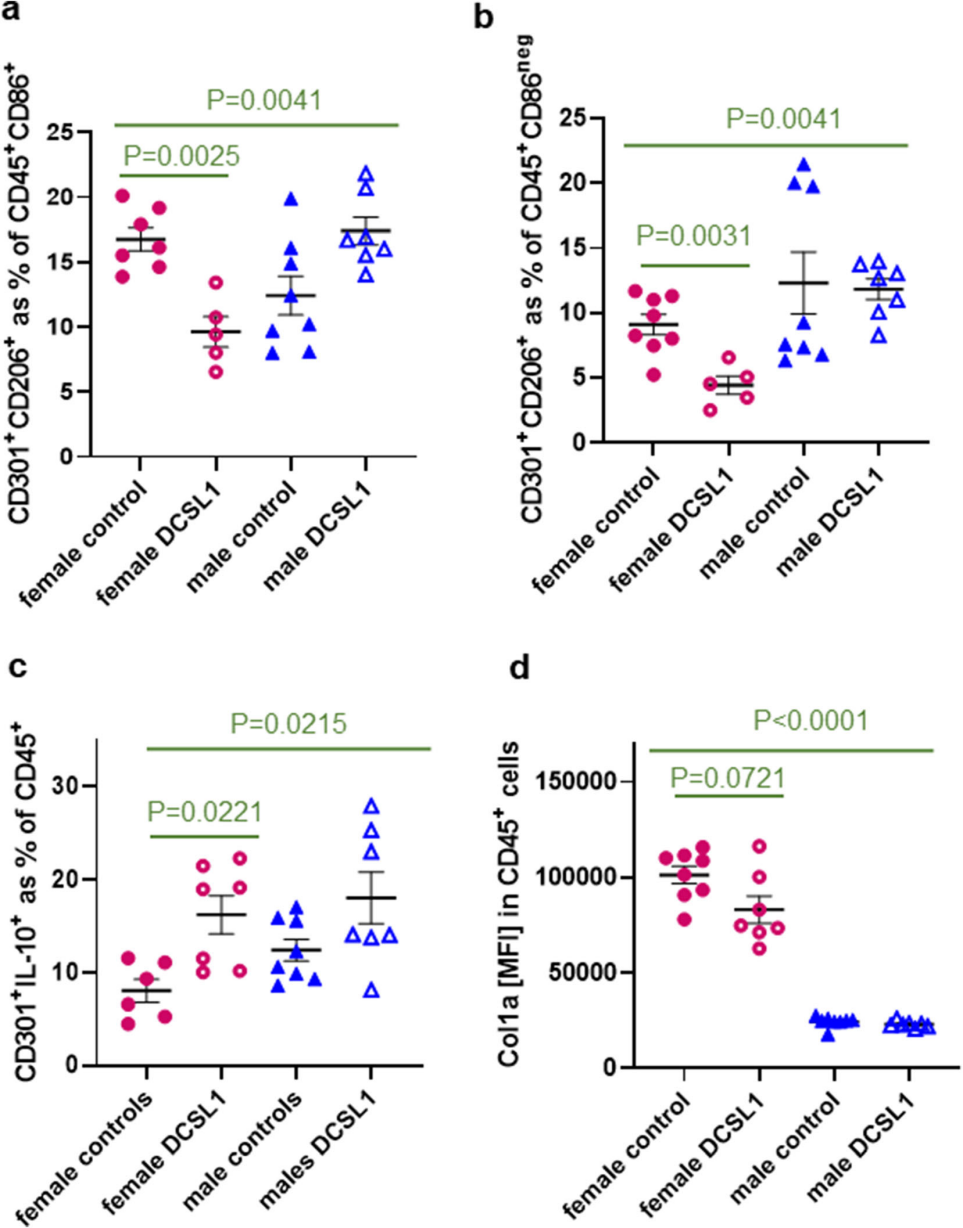
Decrease of pro-fibrotic polarization in female but not in male hearts with DCSL1 treatment (flow cytometry study). **a** The percent of M1/M2 macrophages (CD206^+^CD301^+^) in CD45^+^CD86^+^ leukocytes is reduced in DCSL1-treated female hearts. **b** Polarization towards the pro-fibrotic phenotype defined as the CD206^+^CD301^+^ population in CD45^+^CD86^neg^ cells is decreased in female but not in male hearts. **c** The number of pro-fibrotic macrophages expressing IL-10 (CD45^+^CD301^+^IL-10^+^) was increased by the DCSL1 treatment in the female heart and modestly reduced in the male heart. **d** MFI of collagen type I was slightly reduced by the treatment in female but not in male hearts. For multiple groups, two-way ANOVA was applied with Kruskal-Wallis correction; for only two groups (to compare controls with DCSL1-treated mice), Student’s *t* test followed by the Mann-Whitney test was used as statistical analysis. Results are presented as mean ± SEM. *N* = 5–8 for females and *N* = 7 for males

We then studied the pro-fibrotic differentiation pathway, which is marked by the acquisition of CD206 and CD301 and the loss of CD86. In our previous studies [49], in the human model of transendothelial migration and subsequent macrophage polarization, we reported that the pro-inflammatory CD86^+^ macrophage polarization occurs within the first 16 h after migration. Afterwards, an intermediate phenotype arises that still bore the marker of the inflammatory macrophage (CD86) but had acquired a marker of the pro-fibrotic phenotype (CD206). These cells subsequently developed a mature phenotype defined as CD86^neg^CD206^+^CD301^+^ [49]. In the current mouse experiments, we also found intermediate CD86^+^CD206^+^CD301^+^ macrophages that the DCSL1 treatment reduced by half in the female heart but not in the aged male heart (Fig. 5a). Likewise, the mature pro-fibrotic phenotype identified as the % of CD206^+^CD301^+^ of the CD45^+^CD86^neg^ population was also significantly reduced following DCSL1 treatment in females but not in males (Fig. 5b). Representative images of immunofluorescence staining of CD301 and CD206 in the control and DCSL1-treated female hearts are depicted in Supplemental Fig. 3c.

Our studies in macrophage polarization also found that the DCSL1 treatment triggered a higher number of CD301^+^ macrophages to express IL-10 in the female heart (8.0% in control versus 16.2% in DCSL1-treated) and had a similar (but not statistically significant) effect in males (13.5% in control versus 18.0% in DCSL1-treated) (Fig. 5c). Finally, as another marker of polarized macrophages that we previously defined as pro-fibrotic, we examined the expression of collagen type I alpha (Col1a) [50]. There was a reduction in Col1a mean fluorescence intensity (MFI) in female heart macrophages with DCSL1 treatment (101,192 ± 4557 versus 83,110 ± 7090), but the decrease was not statistically significant (*P* = 0.07). There was no difference in the control and treated male hearts (Fig. 5d).

Consistent with the reduced macrophage polarization in vivo (Figs. 4 and 5), we have found that in the in vitro transendothelial migration assay, DCSL1 greatly reduced the number of spindle-shaped macrophages (“fibrocytes,” Supplemental Fig. 2) even at a very low dose (25 pg/ml).

### DCSL1 treatment affects collagen synthesis and deposition in cardiac tissue

**Fig. 6:**
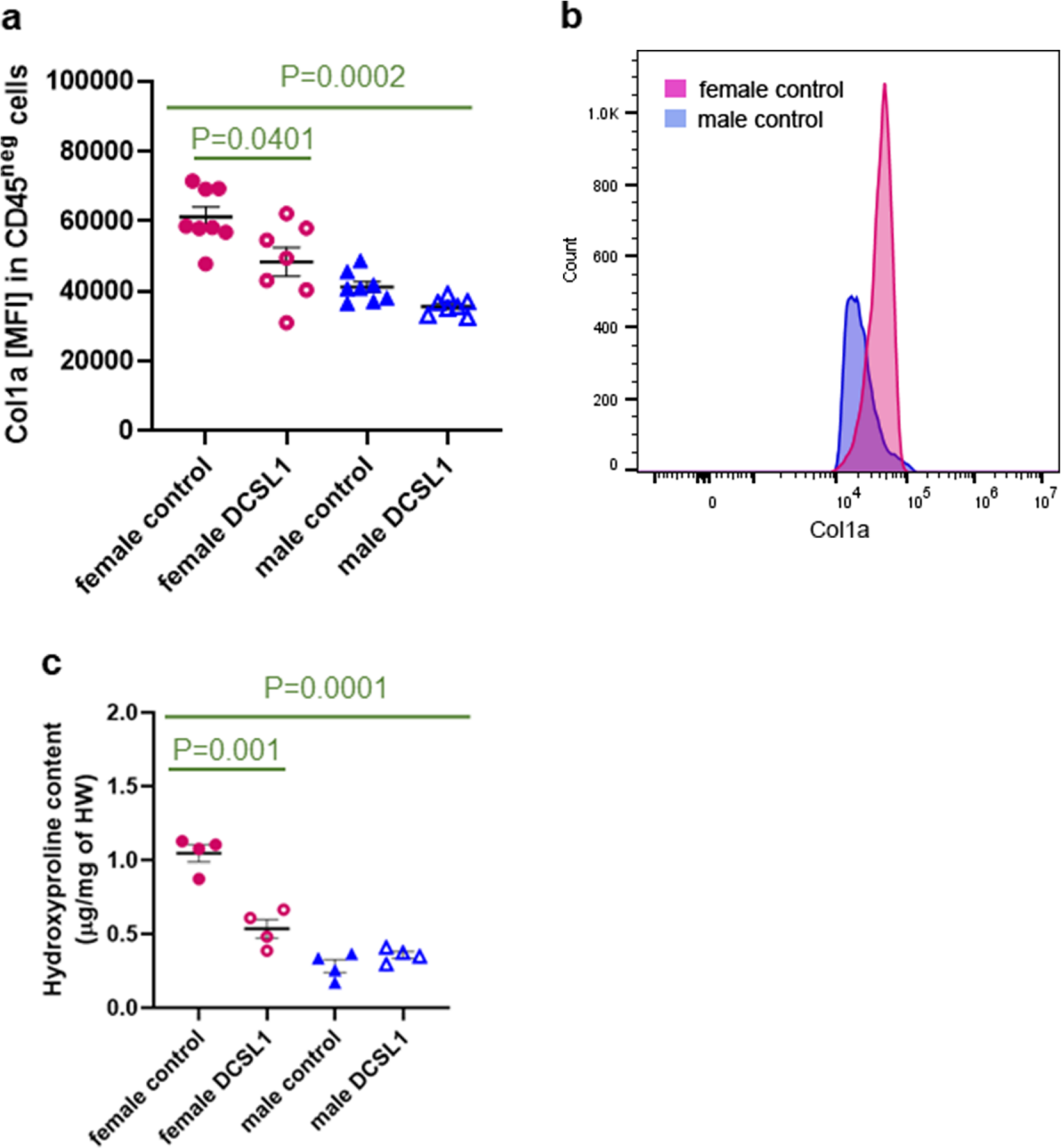
DCSL1 treatment affects collagen synthesis and degradation. **a** Expression of collagen type I in cardiac mesenchymal cells (CD45^neg^) as measured by flow cytometry is affected by DCSL1 treatment in female hearts. **b** Representative histogram of collagen type I expression in CD45^neg^ cells in the female and male hearts as measured by mean fluorescence intensity. **c** Hydroxyproline levels in the control and treated male and female hearts. *N* = 4 animals per group. For multiple groups, two-way ANOVA was applied with Kruskal-Wallis correction; for only two groups (to compare controls with DCSL1-treated mice), Student’s *t* test followed by the Mann-Whitney test was used as statistical analysis

We have previously reported that the reduction of leukocytes seen in aged MCP-1KO mice reduced collagen synthesis in the heart [50]. To determine if a transient treatment with an agent after aging was already underway could be similarly effective, we decided to examine the number of CD45^neg^ (nonleukocyte, including fibroblasts) cells that express Col1a in the control and DCSL1-treated hearts. We found that although the number of CD45^neg^Col1a^+^ cells was unaffected by treatment, nonleukocytes in the control female heart expressed higher levels of collagen than those in the DCSL1-treated female mice (61,206 ± 2878 versus 48,405 ± 4137 MFI) (Fig. 6a). There was no difference in this measure between the control and DCSL1-treated male hearts. Similar to myeloid cells (Fig. 5d), CD45^neg^ cells in female hearts expressed higher levels of collagen than those in males. Figure 6b illustrates the shift in collagen expression between the male and female mesenchymal cells (CD45^neg^Col1a^+^). Next, we assayed the hydroxyproline level in the hearts of the control and treated animals. Hydroxyproline is a major component of collagen protein that plays a role in stabilizing collagen, and its level directly corresponds to the collagen content in tissue. First, we found that hydroxyproline levels were significantly fourfold higher in the control female heart than in the male heart (Fig. 6c), which is consistent with what has been reported previously by others [20] and with data presented in Fig. 6b. We also stained the control male and female hearts with picrosirius red (PSR) and calculated the % of collagen per area (Supplemental Fig. 4a, b). The differences between both sexes resemble the ratio observed with hydroxyproline assay.

Interestingly, and in accordance with our previous results, only DCSL1-treated females exhibited a significant reduction of the level of hydroxyproline (Fig. 6c) which may correlate with the functional improvement we previously found in this group. Similarly, the calculated % of collagen per area (PSR staining) confirmed these data (Supplemental Fig. 4c, d). The proportion of mature and thin fibrils seemed not to be affected by sex or treatment (right lower panel of Supplemental Fig. 4b, d).

**Fig. 7:**
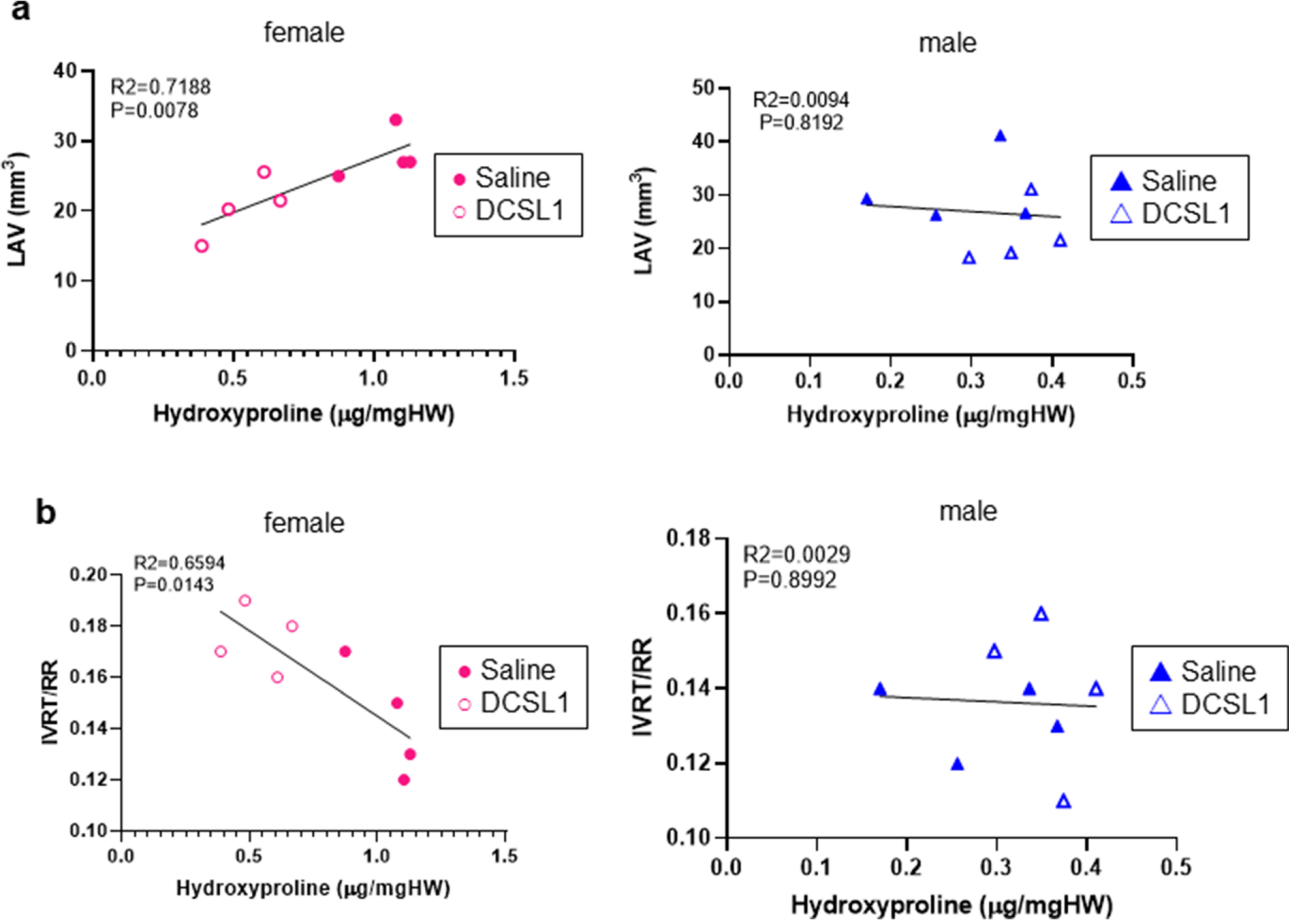
The correlation between heart function parameters and collagen content in the male and female hearts. **a** LAV measurements correlate with hydroxyproline content in the female heart (left panel) but not the male (right panel) heart. **b** The increase in collagen in the females is associated with shortening of IVRT/RR (left panel) but shows no correlation in the male heart (right panel). Student’s *t* test followed by the Mann-Whitney test was used as the statistical analysis, *N* = 4

Next, we explored the relationships between hydroxyproline as a measure of cardiac collagen and two functional parameters that were maintained or improved by DCSL1. The first is LAV (Fig. 7a), which was different between the older females and males. In the females, the LAV was highly correlated with the hydroxyproline content where smaller LAV was associated with decreased hydroxyproline. In males, the levels of hydroxyproline were much lower and there was no relationship between LAV and hydroxyproline. For IVRT/RR, which was increased slightly by DCSL1 and decreased in controls, increased collagen was associated with shorter IVRT. This suggests that “pseudonormalization” as described above was operant over the 3-month period we studied (Fig. 7b).

## Discussion

Even in “healthy” aging there can be interstitial fibrosis [22] and myocardial hypertrophy [30], which can result in abnormal LV relaxation and stiffening. HFpEF in older adults is associated with diastolic dysfunction and has a 2:1 to 3:1 ratio of women to men [6, 18, 32]. Since most studies of the influence of sex on HFpEF incidence and outcome have been retrospective or noninvasive, there has been little ability to investigate the mechanisms responsible [46]. Therefore, animal studies represent an opportunity to test hypotheses and make discoveries concerning specific aspects of the condition. Recent recommendations by the National Institutes of Health that both animal sexes be included as research subjects also supported our decision to study male and female mice. We used natural, intervention-free aging as our subject area, since aging is a risk factor for HFpEF.

Our current discovery that there are cardiac functional differences between the male and female aged mice mirrored the reported differences in humans [3, 39]. Specifically, some aspects of diastolic dysfunction were worse in females than in males (IVRT), but LAV was bigger in males than in females. When we then embarked on a program of following individual mice over a 12-week period of time, we observed a steady decline of function in both males and females. Treatment with the anti-inflammatory drug DCSL1 over the same period of time, in matched cohorts, resulted in different outcomes for males and females. Noninvasive serial measurements of LAV in each individual animal were especially revealing, showing a continued increase in male and female controls as well as treated males. The LAV of DCSL1-treated females did not increase further. The anti-inflammatory intervention with DCSL1 resulted in preserved function for females but not males.

We found that LV mass, which continued to increase with age in both control groups, did not progress in both the male and female treated mice. Yet, the LAV continued to increase in size in treated males without further LV hypertrophy. Therefore, it appears that the increase in LAV and filling pressures can occur without a further increase in LV wall thickness with aging. We found significant differences (*P* = 0.04, Fig. 1a) in response to treatment on LAV between males and females. Specifically, we found that LAV in DCSL1-treated females was unchanged within 12 weeks’ time (heart function did not worsen). By contrast, control females or treated and control males display significantly increased LAV with time corresponding to worsening heart function.

As the diastolic function was impaired at baseline in these old mice, further worsening was associated with paradoxical “improvements” in some of the traditional diastolic parameters, as increased filling pressure leads to increases in *E* peak velocity and shortening of IVRT [37]. The “better” IVRT correlated with increased fibrosis, confirming this interpretation. In contrast, the LAV has a monotonic relationship with diastolic function, and LAV correlated with LV filling pressure and – *dP*/*dt* among old mice and middle-aged mice [34], which have the important limitation that they can only be measured at sacrifice. Because in this study individual changes were followed, the invasive pressure measurements were not possible. Critically all of the mice studied had diastolic dysfunction to varying extents at the onset of the experiments, and we wanted a noninvasive technique that would only get worse as the diastolic function worsened. Additionally, LAV is not a perfect measurement of diastolic function; it can be influenced by a leaky mitral valve or by infiltrative processes in the atrium. However, mitral regurgitation was not observed on the mitral Doppler.

One interpretation of all our data is that the fundamental drivers of age-related diastolic dysfunction are different between the old males and the old females. In the females, fibrosis and collagen appear to be critical and therapeutics targeting fibrotic mechanisms are likely to have benefits. In the males, where the collagen content was lower, targeting fibrosis is less likely to be effective.

Calcium handling proteins implicated in age-related diastolic dysfunction for many decades [21] may play a greater role in males. Interestingly, many of the studies focused on calcium handling use male animals. Importantly, inflammatory processes can drive both impaired calcium handling and fibrosis; our data suggest that fundamentally different approaches may be needed for older people with HFpEF depending on sex.

One aspect of cardiac aging that we previously documented in male mice was the correlation between inflammation and impairment of cardiac function. We observed a reduction of diastolic dysfunction in aging male MCP-1 knockout mice [50]. In the present study, we found no difference in the total leukocyte count from the peripheral blood of males versus females. In contrast, there were striking differences in the numbers and phenotypes of leukocytes in the heart. For example, there were more leukocytes in the male hearts, indicating a higher level of inflammatory infiltration. More of the cells expressing CD45 and CD11b, identifying monocytes, were those highly expressing Ly6C and CCR2 in males versus females. High expression of the markers Ly6C and CCR2 is consistent with monocytes entering the heart after insult or injury [27, 38, 40].

DCSL1 has been reported to be an anti-inflammatory and anti-fibrotic agent, specifically in altering the maturation of monocytes to macrophages [16]. We, therefore, investigated the effect of DCSL1 treatment on the presence and maturation of macrophages in the aging male and female hearts at the end of the 12-week period during which functional studies were also done on each animal. We sought to document changes in the inflammatory state of male versus female myeloid cells residing (transiently or permanently) in the heart and correlated the extent of changes in cardiac function with inflammatory changes in each mouse. We elected not to do lineage tracing because of the difficulty and expense of aging genetically manipulated animals as well as the likelihood that plasticity and replacement of some cell types by others would not yield definitive results.

We found that the macrophage phenotypes previously described in young (usually male) animals were markedly different in aged animals. Categories of macrophages, for example, were originally characterized as M1 through M2 [31, 35] and, although extensively refined and expanded by newer methodologies (reviewed by Murray [36]), can be divided mainly into pro- versus anti-inflammatory. While originally characterized by in vitro differentiation of monocytes, the division of macrophages into pro-inflammatory and anti-inflammatory categories has been confirmed in vivo [23]. We chose to use the simple and accepted method of flow cytometry to determine the status of the cardiac macrophages in our experimental animals. While we found the previously described subclasses of CD86^+^ and CD206^+^ macrophages, a further distinction between the pro-inflammatory subsets, in particular, was striking but more complex. Classically, CD86^+^ macrophages are positive for both pro-inflammatory mediators TNFα and iNOS; in our current studies, CD86^+^ cells were positive for TNFα, iNOS, or both, the proportions of which varied with the sex of the animals and their treatment (see Table 2). One of these subpopulations, CD86^+^TNFα^+^iNOS^neg^ showed marked reduction by DCSL1 treatment in females, but not in males. In addition, CD86^+^ macrophages in males were much more likely to be positive for iNOS than those in females and this was not affected by DCSL1 treatment. Both inflammatory mediators are known to affect cardiac myocytes [9, 26, 41] and so could be the mechanistic link between inflammation and cardiac function.

**Table 2:** Myeloid cell phenotypic changes in response to DCSL1

Cell type	Condition	Response to DCSL1
Monocytes		
Males		
Ratio Ly6C low/high = 1.44	Inflammation	No
CCR2 high > low	New inflammatory infiltrate	No
Females		
Ratio Ly6C low/high = 2.77	Inflammation resolution	No
CCR2 high = low	Chronic inflammatory infiltrate	No
M1 macrophages		
Males		
TNFα^+^iNOS^neg^ (iNOS^+^ males > females)	Inflammation	No
Females		
TNFα^+^iNOS^neg^ (iNOS^+^ females < males)	Inflammation	↓
M2 macrophages		
Males		
CD301^+^CD206^+^ = females	M2 maturation	No
CD301^+^IL-10^+^	Anti-inflammation	=
Females		
CD301^+^CD206^+^ = males	M2 maturation	↓
CD301^+^IL-10^+^	Anti-inflammation	↑

The total number of macrophages that responded to DCSL1 treatment and expressed TNFα is about 1/3 of the total leukocytes (in Fig. 4, these cells are presented as a percent of the CD45^+^CD86^+^ subpopulation). Because these cells produce soluble, secreted cytokines, they are difficult to detect by immunofluorescence staining in sections (however, we included some staining in the Supplemental Fig. 3b). To be able to identify them by flow cytometry, we incubated cells with brefeldin and monensin, inhibitors of the protein transport from the endoplasmic reticulum to the Golgi. This manipulation allows the buildup of the cytokine levels in the cytoplasm and easier detection.

Similar effects of DCSL1 in females but not males were found in other macrophages. We reported that in vitro human monocytes maturing after transendothelial migration acquired first CD86, then added CD206, and eventually added CD301 before dropping the expression of CD86 [49]. Macrophages with mixed phenotypes have been found in the injury and disease states [10, 47]. In our study, there were mixed populations in the hearts of aged mice, and these were differentially affected by DCSL1 treatment between the two sexes. In females, cells with CD86, CD206, and CD301 (as a percent of all CD45^+^), as well as CD86^neg^CD206^+^CD301^+^ mature macrophages, were decreased by DCSL1. The same populations in males were not affected by treatment.

The macrophages bearing a mature pro-fibrotic phenotype (CD301) were also different with respect to their response to DCSL1 treatment; CD301^+^IL-10^+^ macrophages more than doubled their numbers in females, but in males, they were not increased significantly by DCSL1. The anti-inflammatory cytokine IL-10 can deactivate macrophages, suppressing TNFα release as a result [7], which may explain the drop in numbers of inflammatory macrophages making TNFα in the treated females.

DC-SIGN is a carbohydrate-binding receptor that recognizes pathogens via pathogen-associated molecular patterns as part of innate immunity. Ligation of DC-SIGN on human monocytes results in a tolerogenic potential, with increased IL-10 production [44]. It also decreases the pro-fibrotic cytokine IL-4 signaling via pSTAT6, perhaps resulting in the decreased fibrosis that others have reported in a mouse disease model [16]. In the aging mouse heart, both an IL-10 increase and pro-fibrotic phenotype decrease occurred in the cardiac macrophages of females treated with the ligand DCSL1. Although we do not have a direct connection between the observed decrease in TNFα^+^ macrophages and the decrease in pro-fibrotic macrophages in the old females, we previously published that TNFα production was necessary for the maturation of pro-fibrotic macrophages in young animals [33]. In turn, the decrease in TNFα^+^ cells in the old females may have been due to the increase in IL-10^+^ macrophages initiated by exposure to DCSL1.

We also analyzed T cells to determine if their phenotype was affected by DCSL1, which might have directed subsequent macrophage polarization. Among T lymphocytes, only the total numbers of CD3^+^ in males were altered by DCSL1. However, there were sex differences in untreated subpopulations of T cells. The ratio of CD4^+^ to CD8^+^ T cells in the hearts of the old mice ranged from 0.08 (in males) to 0.34 (in females). It is possible that the higher numbers of CD4^+^ T cells in the female hearts may be related to a reported role for these cells in heart failure [2], especially diastolic dysfunction [53]. A high number of CXCR3^+^CD8^+^ T cells were noted in male hearts, but not in females. These cells are reported to have a naïve phenotype in aging that can promote inflammation [29]. Their presence in the aged male heart could be responsible for the increased inflammatory macrophage polarization in males. All these suggest that although T cells per se are not directly affected by DCSL1 treatment, the sex differences seen in their phenotype may be significant in the therapeutic outcome for other cells such as macrophages.

Consistent with this connection with fibrosis, nonleukocytes had lower amounts of collagen type I in their cytoplasm after DCSL1 treatment in females. Untreated males had lower amounts of collagen in all cell types, which were not affected by DCSL1 treatment. In females, treatment was accompanied by a reduction of hydroxyproline levels in the heart to the lower levels found in young female hearts.

Activated fibroblasts in the aging heart contribute not only to fibrosis but also to inflammation, which may be due, in part, to mitochondrial stress. A recent study suggests that by optimizing mitochondrial function in fibroblast and normalizing their metabolism by using rapamycin, they can resist senescence [17]. Therefore, an optimized method to measure respiratory function in the isolated cardiac mitochondria may provide a useful tool to examine the role of mitochondrial function in several aspects of fibroblast physiology or rescue experiments [52].

To summarize, both the male and female hearts develop a chronic inflammatory response with aging, but the characteristics of the response were markedly different in each sex. The males had a TNFα/iNOS and IL-10 immune response in the heart, which was not affected by DCSL1 treatment. Females had a TNFα response, which was reduced by DCSL1, possibly through upregulation of IL-10. The pro-fibrotic macrophages in females, which we reported to contribute to fibrosis via communication with fibroblasts [50], were likewise decreased by DCSL1. The leukocyte changes were associated with a preservation of cardiac function in the DCSL1-treated females while deterioration continued in the other groups. Correlating progression over a 3-month period allowed assessment of therapeutic efficacy in each mouse. The results strikingly resemble the increased risk for the clinical counterpart of this model in female patients in our aging population. They provide the potential for the development of targeted therapeutic interventions and underscore the need to characterize the diverse phenotypes of HFpEF.

The importance of sex in the approach to HFpEF and diastolic dysfunction of aging must be emphasized. The cardiomyocyte hypertrophy in response to age and to loading is modified by sex in animal models as well as in humans [39]. The inflammatory milieu is also different in older women compared with older men [3]. One manifestation of this sex effect was seen in the PARAGON-HF trial, a large study of angiotensin–neprilysin inhibition in patients with HFpEF. The benefit of treatment was seen only in the women in the study, not in the men [41]. As HFpEF is predominantly a disease of older women, females must be included in any clinical or translational effort to ensure that the results are directly relevant to the target population [28].

## Electronic supplementary material

ESM 1 (DOCX 532 kb)
