# Supplementary material for: Treatment with a DC-SIGN ligand reduces macrophage polarization and diastolic dysfunction in the aging female but not male mouse hearts
Source: GeroScience. 2020 Aug 26;43(2):881–99. doi: 10.1007/s11357-020-00255-4 (PMC8110645; doi:10.1007/s11357-020-00255-4)
Supplement: Supplementary file 1 — (DOCX 532 kb) [file 11357_2020_255_MOESM1_ESM.docx]

**SUPPLEMENTAL MATERIAL**

**Treatment with a DC-SIGN ligand reduces macrophage polarization and diastolic dysfunction in the aging female but not male mouse heart**

**Journal: Geroscience**

JoAnn Trial^1^, Rodrigo Diaz Lankenau^1^, Aude Angelini^1^, Jorge E. Tovar Perez^1,2^, George E. Taffet^1,3^, Mark L. Entman^1,3^, and Katarzyna A. Cieslik^1^*

^1^ Department of Medicine, Cardiovascular Research, Baylor College of Medicine, One Baylor Plaza, MS: BCM 620, Houston, TX 77030, USA; ^2^ current address: Texas A&M University, 2121 W. Holcombe Blvd, Houston, TX 77030, USA; ^3^ the DeBakey Heart Center, Houston Methodist Hospital, 6565 Fannin Street, Houston, TX 77030, USA; * corresponding author

Corresponding author:

Dr. Katarzyna A. Cieslik

Email: [cieslik@bcm.edu](mailto:cieslik@bcm.edu)

**Supplemental Methods**

Blood leukocyte count - To estimate the number of circulating CD45^+^ cells, we collected blood directly from the left ventricle. An aliquot of 50µL was collected into a K_2_EDTA tube (BD Biosciences, #365974)) for this assay. Blood cells were directly labeled with anti-CD45 antibody (Biolegend #109828). Red blood cells were lysed by adding 450µl of red cell lysis buffer (Biolegend # 420302) for 20 minutes at 4°C, then any clumps were removed by passing the cell suspension through a 40µm strainer. This protocol was adapted from a report by Brunck et al [[1](#_ENREF_1)]. The suspension of white blood cells was diluted at 1:8 in a flow buffer (DPBS with calcium and magnesium, 0.1% sodium azide and 0.1% gelatin) and counted on a CytoFlex flow cytometer (Beckman Coulter). Acquired data were analyzed using CytoExpert 2.3 software (Beckman Coulter).

Transendothelial migration- As previously reported [[3](#_ENREF_3)] human cardiac microvascular cells (ScienCell, #6000) were grown on a membrane with eight micron pores (Costar Transwell, Corning, #3422). Blood was obtained from healthy volunteers under a protocol approved by the Institutional Review Board of Baylor College of Medicine. Mononuclear cells from these donors were added to each insert after endothelial cells reached full confluence. Leukocytes transmigrated through the endothelial barrier in response to chemoattractant (640 ng/ml of CCL2, R&D Systems, #279-MC). Medium (RPMI1640 with 10% FBS) below the insert also contained an M2 polarization enhancer (10 ng/ml of IL-13, R&D Systems, #213-IL). The transmigrated monocytes with a spindle (fibrocyte) morphology were counted at the end of four days. The control was set up in the same way, but without chemoattractant or enhancer below the endothelial cells. Other cultures, with CCL2 and IL-13, were treated with the indicated amount of DCSL1 during the entire four days.

Immunofluorescence microscopy- Paraffin-embedded zinc-Tris fixed mouse heart sections (mid sections) were incubated overnight with the following antibodies: CD45 (Biolegend, #103122), TNF-α (Biolegend, #506345), iNOS (Biolegend, # 696803), CD301 (Biolegend, #145704), and CD206 (Biolegend, #141704).

Picrosirius red staining and polarized light assessment of fibrillar collagen- Cross-sections of ventricles (mid sections) were stained with picrosirius red (PolyScientiifc, #S2365) [[2](#_ENREF_2)] with a 0.01N HCl wash [[4](#_ENREF_4)]. The sections were examined under brightfield illumination with an Olympus CKX41 or under an Olympus AX70 microscope equipped with filters for linear polarization [[4](#_ENREF_4)]. The polarizer was rotated to achieve extinction with the analyzer, showing a black background and the birefringency of the picrosirius red-stained collagen. Images were analyzed using ImageJ software. The RGB images were converted to an HSB (hue, saturation, brightness) stack and from those were obtained histograms of the hue spectrum that were quantified for each hue after setting the spectral limits for red, orange, yellow, and green. Biological repeats n=3 for treated and control animals, respectively, experimental repeats n=3.

1. Brunck ME, Andersen SB, Timmins NE, Osborne GW, Nielsen LK (2014) Absolute counting of neutrophils in whole blood using flow cytometry. Cytometry A 85:1057-1064 doi:10.1002/cyto.a.22503

2. Constantine VS, Mowry RW (1968) Selective staining of human dermal collagen. II. The use of picrosirius red F3BA with polarization microscopy. J Invest Dermatol 50:419-423

3. Haudek SB, Gupta D, Dewald O, Schwartz RJ, Wei L, Trial J, Entman ML (2009) Rho kinase-1 mediates cardiac fibrosis by regulating fibroblast precursor cell differentiation. Cardiovasc Res 83:511-518 doi:10.1093/cvr/cvp135

4. Junqueira LC, Cossermelli W, Brentani R (1978) Differential staining of collagens type I, II and III by Sirius Red and polarization microscopy. Arch Histol Jpn 41:267-274

Supplemental Table 1. Antibodies used for flow cytometry.

| **Target antigen** | **Vendor** | **Catalog number** | **Clone** | **Dilution** |
| --- | --- | --- | --- | --- |
| CD45 | Biolegend | #109828 | 104 | 1:50 |
| CD11b | Invitrogen | #12-0112-82 | M1/70 | 1:166 |
| CCR2 | Biolegend | #150612 | SA203G11 | 1:20 |
| CX3CR1 | Biolegend | #149035 | SA011F11 | 1:200 |
| Ly6C | Biolegend | #128010 | HK1.4 | 1:100 |
| iNOS | Invitrogen | #17-5920-80 | CXNFT | 1:333 |
| TNFα | Biolegend | #506345 | MP6-XT22 | 1:166 |
| CD86 | Biolegend | #105024 | GL-1 | 1:50 |
| CD301 | Biolegend | #145704 | LOM-14 | 1:100 |
| CD206 | Biolegend | #141710 | C068C2 | 1:50 |
| IL-10 | Biolegend | #505034 | JES5-16E3 | 1:33 |
| Col1a | Rockland Immunochemicals | #600-401-103 |  | 1:100 |
| CD16/CD32 | Biolegend | #101320 | 93 | 1:20 |
| Anti-rabbit IgG | Jackson ImmunoResearch | #711-675-152 |  | 1:100 |
| CD3 | Biolegend | #100218 | 17A2 | 1:58 |
| CD56 | Novus Biologicals | #NBP2-52710 | 735 | 1:100 |
| CD4 | Biolegend | #100512 | RM4-5 | 1:80 |
| CD8a | Biolegend | #100762 | 53-6.7 | 1:66 |
| CXCR3 | Biolegend | #566283 | CXCR3-173 | 1:50 |
| CD103 | Biolegend | #121426 | 2E7 | 1:31 |
| CD11c | Biolegend | #117324 | N418 | 1:20 |

Supplemental Table 2a. Echo measurements in 3-month-old and 21-month-old females.

|  | **Young** | **Old** | **T-Test** |
| --- | --- | --- | --- |
| **LA AP (mm)** | 1.99 ± 0.09 | 2.27 ± 0.07 | 0.03629 |
| **LVAW;d (mm)** | 0.61 ± 0.05 | 0.88 ± 0.03 | 0.00038 |
| **LVAW;s (mm)** | 1.02 ± 0.07 | 1.29 ± 0.05 | 0.00574 |
| **Diameter;d (mm)** | 4.25 ± 0.02 | 4.04 ± 0.06 | ns |
| **Diameter;s (mm)** | 3.11 ± 0.05 | 2.80 ± 0.06 | 0.01280 |
| **Volume;d (μl)** | 79.3 ± 1.7 | 71.6 ± 2.3 | ns |
| **Volume;s (μl)** | 38.3 ± 1.6 | 32.2 ± 1.7 | ns |
| **LVPW;d (mm)** | 0.78 ± 0.04 | 1.04 ± 0.05 | 0.01014 |
| **LVPW;s (mm)** | 0.98 ± 0.06 | 1.33 ± 0.06 | 0.00331 |
| **Stroke Volume (μl)** | 42.4 ± 1.2 | 41.8 ± 1.2 | ns |
| **Ejection Fraction (%)** | 52.5 ± 1.4 | 58.5 ± 1.3 | 0.01845 |
| **Fractional Shortening (%)** | 26.8 ± 0.9 | 30.8 ± 0.9 | 0.02262 |
| **Cardiac Output (ml/min)** | 15.0 ± 0.8 | 16.7 ± 0.6 | ns |
| **Relative Wall Thickness** | 0.37 ± 0.02 | 0.52 ± 0.03 | 0.00663 |
| **LV Mass (AW) Corrected (mg)** | 86 ± 5.6 | 123 ± 5.2 | 0.00074 |
| **LA SI (mm)** | 5.70 ± 0.08 | 5.67 ± 0.07 | ns |
| **LA ML (mm)** | 2.26 ± 0.04 | 2.53 ± 0.05 | 0.00437 |
| **Aorta (mm)** | 1.44 ± 0.02 | 1.54 ± 0.01 | 0.00023 |
| **Pulmonary Artery (mm)** | 1.40 ± 0.03 | 1.52 ± 0.02 | 0.00502 |
| **LAA (ML&SI) (mm^2^)** | 10.1 ± 0.2 | 11.3 ± 0.3 | 0.02173 |
| **LAA (AP&ML) (mm^2^)** | 3.5 ± 0.2 | 4.5 ± 0.2 | 0.00628 |
| **LAV (mm^3^)** | 13.4 ± 0.7 | 17.3 ± 0.8 | 0.01965 |
| **PAca (mm^2^)** | 1.54 ± 0.1 | 1.82 ± 0 | 0.00540 |
| **LAV/PAca (mm)** | 8.7 ± 0.3 | 9.7 ± 0.5 | ns |
| **Body Weight (g)** | 23.0 ± 0.3 | 30.6 ± 0.8 | 0.00001 |
| **Body Surface Area (mm^2^)** | 7272 ± 73 | 8794 ± 154 | 0.00001 |

**Abbreviations**: LA AP: Left atrium anteroposterior diameter; LVAW;d: Left ventricle anterior wall diameter during diastole; LVAW;s: Left ventricle anterior wall diameter during systole; Diameter;d: Left ventricle interior diameter during diastole; Diameter;s: Left ventricle interior diameter during systole; Volume;d: Left ventricle interior volume during diastole; Volume;s: Left ventricle interior volume during systole; LVPW;d: Left ventricle posterior wall diameter during diastole; LVPW;s: Left ventricle posterior wall diameter during systole; LV mass (AW): Left ventricle mass (Anterior wall); LA SI: Left atrium superoinferior diameter; LA ML: Left atrium mediolateral diameter; LAV: Left atrium volume; PAca: Pulmonary artery; LAV/PAca: Left atrial volume/Pulmonary artery; ns: not statistically significant.

Supplemental Table 2b. Doppler measurements in 3-month-old and 21-month-old females.

|  | **Young** | **Old** | **T-Test** |
| --- | --- | --- | --- |
| **Heart Rate (bpm)** | 451 ± 13 | 465 ± 8 | ns |
| **E-Peak Velocity (mm/s)** | 738 ± 14 | 634 ± 18 | 0.00392 |
| **E-Stroke Distance (mm)** | 12.2 ± 0.4 | 10.6 ± 0.4 | 0.04925 |
| **E-Time Duration (ms)** | 22.5 ± 1 | 21.5 ± 0.5 | ns |
| **E-Peak to ½E-Peak Time** | 24 ± 1.8 | 22.4 ± 1.3 | ns |
| **E-Linear Deceleration Time (ms)** | 48 ± 3.5 | 44.8 ± 2.6 | ns |
| **E-Linear Deceleration Rate** | 1669 ± 171 | 1574 ± 98 | ns |
| **A-Peak Velocity (mm/s)** | 534 ± 20 | 493 ± 14 | ns |
| **A-Stroke Distance (mm)** | 10.7 ± 0.4 | 9 ± 0.3 | 0.01049 |
| **A-Time Duration (ms)** | 30.3 ± 1.6 | 25 ± 0.8 | 0.00594 |
| **E-A Peak Velocity Ratio** | 1.4 ± 0.1 | 1.29 ± 0 | 0.03971 |
| **Isovolumic Contraction Time (ms)** | 13.9 ± 0.9 | 15.7 ± 0.7 | ns |
| **Isovolumic Relaxation Time (ms)** | 17.6 ± 0.9 | 21.4 ± 0.7 | 0.00771 |
| **IVRT/RR** | 0.13 ± 0 | 0.16 ± 0 | 0.00002 |
| **Pre-ejection Time (ms)** | 18.6 ± 0.7 | 18.5 ± 0.5 | ns |
| **Peak Aortic Velocity (mm/s)** | 898 ± 64 | 755 ± 20 | 0.00975 |
| **Stroke Distance (mm)** | 33.4 ± 2.4 | 25.3 ± 0.8 | 0.00036 |
| **Ejection Time (ms)** | 50.8 ± 1.5 | 48.4 ± 1 | ns |
| **Rise Time (ms)** | 14.3 ± 0.7 | 13.3 ± 0.3 | ns |
| **Mean Velocity (mm/s)** | 248 ± 20 | 194 ± 7 | 0.00284 |
| **Mean Acceleration (mm/s^2^)** | 66342 ± 6422 | 60203 ± 2368 | ns |
| **Peak Acceleration (mm/s^2^)** | 206628 ± 15782 | 173615 ±13433 | ns |

**Abbreviations**: E-peak velocity: Early-wave peak velocity; E-stroke distance: Early-wave stroke distance; E-time duration: Early-wave time duration; E-Peak to ½E-Peak time: Time between Early-wave peak velocity and ½Early-wave peak velocity; E-Linear deceleration time: Early-wave linear deceleration time; E-linear deceleration rate: Early-wave deceleration rate; A-peak velocity: Atrial-wave peak velocity; A-stroke distance: Atrial-wave stroke distance; A-time duration: Atrial-wave time duration; E-A peak velocity ratio: Early-wave/Atrial-wave peak velocity ratio; IVRT/RR: Isovolumic relaxation time/RR-Interval ratio; ns: not statistically significant.

Supplemental Table 3a. Echo measurements in 21-month-old females and males.

|  | **Females** | **Males** | **T-Test** |
| --- | --- | --- | --- |
| **LA AP (mm)** | 2.33 ± 0.1 | 2.46 ± 0.1 | ns |
| **LVAW;d (mm)** | 0.89 ± 0.04 | 1.05 ± 0.04 | 0.01012 |
| **LVAW;s (mm)** | 1.31 ± 0.1 | 1.44 ± 0.1 | ns |
| **Diameter;d (mm)** | 4.04 ± 0.1 | 4.22 ± 0.1 | ns |
| **Diameter;s (mm)** | 2.8 ± 0.1 | 3.0 ± 0.1 | ns |
| **Volume;d (μl)** | 72.2 ± 2.4 | 80.3 ± 3.8 | ns |
| **Volume;s (μl)** | 30.4 ± 1.6 | 36.2 ± 2.8 | ns |
| **LVPW;d (mm)** | 1.06 ± 0.1 | 1.06 ± 0.04 | ns |
| **LVPW;s (mm)** | 1.35 ± 0.1 | 1.36 ± 0.1 | ns |
| **Stroke Volume (μl)** | 41.8 ± 1.3 | 44.1 ± 1.6 | ns |
| **Ejection Fraction (%)** | 58.5 ± 1.2 | 55.9 ± 1.6 | ns |
| **Fractional Shortening (%)** | 30.7 ± 0.9 | 29.1 ± 1.0 | ns |
| **Cardiac Output (ml/min)** | 16.8 ± 0.6 | 17.9 ± 0.8 | ns |
| **Relative Wall Thickness** | 0.53 ± 0.03 | 0.52 ± 0.03 | ns |
| **LV Mass (AW) Corrected (mg)** | 126 ± 5.3 | 148 ± 5.6 | 0.00867 |
| **Heart Rate (bpm)** | 427 ± 11 | 443 ± 13 | ns |
| **LA SI (mm)** | 5.66 ± 0.1 | 5.96 ± 0.1 | 0.00669 |
| **LA ML (mm)** | 2.58 ± 0.1 | 2.73 ± 0.1 | ns |
| **Aorta (mm)** | 1.54 ± 0.01 | 1.66 ± 0.02 | 0.00001 |
| **Pulmonary Artery (mm)** | 1.52 ± 0.02 | 1.44 ± 0.03 | ns |
| **LAV (mm^3^)** | 18.1 ± 1.0 | 21.7 ± 1.1 | 0.01856 |
| **PAca (mm^2^)** | 1.8 ± 0.1 | 1.64 ± 0.1 | ns |
| **LAV/PAca (mm)** | 10.3 ± 0.6 | 13.5 ± 0.8 | 0.00183 |
| **Body Weight (g)** | 30.6 ± 0.8 | 36 ± 0.9 | 0.00006 |
| **Body Surface Area (mm^2^)** | 8794 ± 154 | 9806 ± 156 | 0.00006 |

**Abbreviations**: LA AP: Left atrium anteroposterior diameter; LVAW;d: Left ventricle anterior wall diameter during diastole; LVAW;s: Left ventricle anterior wall diameter during systole; Diameter;d: Left ventricle interior diameter during diastole; Diameter;s: Left ventricle interior diameter during systole; Volume;d: Left ventricle interior volume during diastole; Volume;s: Left ventricle interior volume during systole; LVPW;d: Left ventricle posterior wall diameter during diastole; LVPW;s: Left ventricle posterior wall diameter during systole; LV mass (AW): Left ventricle mass (Anterior wall); LA SI: Left atrium superoinferior diameter; LA ML: Left atrium mediolateral diameter; LAV: Left atrium volume; PAca: Pulmonary artery; LAV/PAca: Left atrial volume/Pulmonary artery; ns: not statistically significant.

Supplemental Table 3b. Doppler indices in 21-month-old females and males.

|  | **Females** | **Males** | **T-Test** |
| --- | --- | --- | --- |
| **Heart Rate (bpm)** | 465 ± 8 | 437 ± 12 | ns |
| **E-Peak Velocity (mm/s)** | 634 ± 18 | 624 ± 15 | ns |
| **E-Stroke Distance (mm)** | 10.6 ± 0.4 | 12.0 ± 0.4 | 0.02215 |
| **E-Time Duration (ms)** | 21.5 ± 0.5 | 29.7 ± 1.8 | 0.00001 |
| **E-Peak to ½E-Peak Time** | 22.4 ± 1.3 | 18.2 ± 1.6 | 0.04634 |
| **E-Linear Deceleration Time (ms)** | 44.9 ± 2.6 | 36.5 ± 3.1 | 0.04633 |
| **E-Linear Deceleration Rate** | 1574 ± 98 | 2016 ± 164 | 0.02124 |
| **A-Peak Velocity (mm/s)** | 493 ± 14 | 452 ± 24 | ns |
| **A-Stroke Distance (mm)** | 9.0 ± 0.3 | 9.5 ± 0.4 | ns |
| **A-Time Duration (ms)** | 25 ± 0.8 | 30.2 ± 1.0 | 0.00024 |
| **E-A Peak Velocity Ratio** | 1.29 ± 0.02 | 1.48 ± 0.1 | ns |
| **Isovolumic Contraction Time (ms)** | 15.7 ± 0.7 | 16.1 ± 1.0 | ns |
| **Isovolumic Relaxation Time (ms)** | 21.4 ± 0.7 | 18.4 ± 0.5 | 0.00169 |
| **IVRT/RR** | 0.16 ± 0.003 | 0.13 ± 0.004 | <0.00001 |
| **Pre-ejection Time (ms)** | 18.5 ± 0.5 | 16.8 ± 0.5 | 0.02703 |
| **Peak Aortic Velocity (mm/s)** | 755 ± 20 | 933 ± 27 | <0.00001 |
| **Stroke Distance (mm)** | 25.3 ± 0.8 | 30.4 ± 1.3 | 0.00179 |
| **Ejection Time (ms)** | 48.4 ± 1.0 | 44.9 ± 1.0 | 0.02133 |
| **Rise Time (ms)** | 13.3 ± 0.3 | 14 ± 0.6 | ns |
| **Mean Velocity (mm/s)** | 194 ± 6.7 | 227 ± 8.5 | 0.00460 |
| **Mean Acceleration (mm/s^2^)** | 60203 ± 2368 | 70547 ± 2818 | 0.00811 |
| **Peak Acceleration (mm/s^2^)** | 173615±13434 | 197806±16774 | ns |

**Abbreviations**: E-peak velocity: Early-wave peak velocity; E-stroke distance: Early-wave stroke distance; E-time duration: Early-wave time duration; E-Peak to ½E-Peak time: Time between Early-wave peak velocity and ½Early-wave peak velocity; E-Linear deceleration time: Early-wave linear deceleration time; E-linear deceleration rate: Early-wave deceleration rate; A-peak velocity: Atrial-wave peak velocity; A-stroke distance: Atrial-wave stroke distance; A-time duration: Atrial-wave time duration; E-A peak velocity ratio: Early-wave/Atrial-wave peak velocity ratio; IVRT/RR: Isovolumic relaxation time/RR-Interval ratio; ns: not statistically significant.

**Supplemental Table 4a. The effect of DCSL1 on female hearts.**

|  | **Controls** | | | **DCSL1** | | | **T-Test** | |
| --- | --- | --- | --- | --- | --- | --- | --- | --- |
|  | **Baseline** | **% of change** | | **Baseline** | **% of change** | |  |  |
|  |  | **4 weeks** | **12 weeks** |  | **4 weeks** | **12 weeks** | **4 weeks** | **12 weeks** |
| **HR (bpm)** | 427 ± 19 | 16.5% ± 5% | 20.5% ± 9% | 426 ± 11 | 3.9% ± 5% | 8.8% ± 5% | ns | ns |
| **Volume;d (μl)** | 69.7 ± 3.1 | 8.1% ± 4% | 11.1% ± 7% | 73.3 ± 3.2 | 13.9% ± 2% | -6.5% ± 5% | ns | ns |
| **Volume;s (μl)** | 29.4 ± 2.4 | 15.8% ± 10% | 20% ± 15% | 34.7 ± 2.1 | 15.9% ± 6% | -12.2% ± 7% | ns | ns |
| **Stroke volume (μl)** | 40.3 ± 1.5 | 6.5% ± 5% | 6.8% ± 7% | 38.6 ± 1.8 | 14.2% ± 4% | -0.8% ± 6% | ns | ns |
| **EF (%)** | 58.5 ± 2 | -1.3% ± 4% | -2.7% ± 6% | 52.9 ± 1.7 | 0.5% ± 4% | 6.6% ± 3% | ns | ns |
| **FS (%)** | 30.7 ± 1.3 | -1.4% ± 5% | -2.7% ± 7% | 27.0 ± 1.1 | 1.5% ± 5% | 8.5% ± 4% | ns | ns |
| **CO (ml/min)** | 17.2 ± 1.0 | 24.4% ± 8% | 26.9% ± 9% | 16.4 ± 0.8 | 18.7% ± 7% | 7.5% ± 7% | ns | ns |
| **LVAW;d (mm)** | 0.86 ± 0.1 | 5.3% ± 11% | 24.1% ± 10% | 0.89 ± 0.03 | -10.4% ± 5% | 10.8% ± 5% | ns | ns |
| **LVAW;s (mm)** | 1.27 ± 0.1 | 8.1% ± 12% | 27% ± 12% | 1.32 ± 0.04 | -5.8% ± 6% | 8.9% ± 5% | ns | ns |
| **LVPW;d (mm)** | 1.07 ± 0.1 | 0.3% ± 7% | 9.0% ± 5% | 1.01 ± 0.1 | 2.9% ± 7% | 1.5% ± 7% | ns | ns |
| **LVPW;s (mm)** | 1.44 ± 0.1 | 2.5% ± 8% | -1.8% ± 2% | 1.24 ± 0.1 | 1.7% ± 6% | 5.3% ± 5% | ns | ns |
| **LV Mass (AW) Corrected** | 122 ± 8.5 | 5.9% ± 5% | 27.6% ± 7% | 124 ± 6.3 | 1.8% ± 6% | -0.3% ± 4% | ns | 0.00273 |
| **LV Mass (Teichholz)**[**3**](#_ENREF_1) | 87.6 ± 8.2 | 3.8% ± 9% | 14.0% ± 5% | 83.1 ± 7.1 | 8.9% ± 9% | -1.1% ± 7% | ns | ns |
| **LA AP (mm)** | 2.24 ± 0.1 | -1.2% ± 5% | 12.0% ± 6% | 2.29 ± 0.1 | 1.5% ± 4% | -3.5% ± 3% | ns | ns |
| **LA ML (mm)** | 2.57 ± 0.1 | 7.6% ± 3% | 11.6% ± 5% | 2.50 ± 0.1 | 4.8% ± 3% | -0.5% ± 2% | ns | 0.02208 |
| **LA SI (mm)** | 5.75 ± 0.1 | 0.5% ± 2% | 5.8% ± 3% | 5.61 ± 0.1 | 0.4% ± 2% | 9.2% ± 2% | ns | ns |
| **LA V (mm^3^)** | 17.5 ± 1.2 | 6.3% ± 5% | 31.3% ± 8% | 17.0 ± 1.2 | 6.9% ± 6% | 5.4% ± 6% | ns | 0.02152 |
| **E peak velocity (mm/s)** | 645 ± 19 | 2.1% ± 3% | 9.5% ± 7% | 624 ± 29 | 4.6% ± 6% | 7.9% ± 6% | ns | ns |
| **A peak velocity (mm/s)** | 507 ± 13 | 2.4% ± 5% | 6.3% ± 6% | 480 ± 24 | 8.9% ± 7% | 19.1% ± 6% | ns | ns |
| **E-A peak velocity ratio** | 1.28 ± 0.03 | 2.8% ± 7% | 4.5% ± 7% | 1.31 ± 0.03 | -3.0% ± 2% | -9.4% ± 2% | ns | 0.04399 |
| **IVCT (ms)** | 15.3 ± 0.8 | -20.5% ± 5% | -6.7% ± 9% | 16.0 ± 1.1 | -8.5% ± 9% | -3.9% ± 9% | ns | ns |
| **IVRT (ms)** | 19.6 ± 0.6 | -4.9% ± 4% | -6.8% ± 8% | 23.0 ± 1 | -16% ± 4% | -9.6% ± 7% | ns | ns |
| **IVRT/RR** | 0.16 ± 0.01 | -11.3% ± 3% | -7.8% ± 7% | 0.17 ± 0.004 | -14.4% ± 3% | -1.4% ± 6% | ns | ns |
| **Aortic peak velocity (mm/s)** | 776 ± 23 | 15.9% ± 5% | 11.9% ± 4% | 736 ± 32 | 21.1% ± 5% | 16.4% ± 6% | ns | ns |
| **LVM/BSA (mg/cm^2^)** | 1.24 ±0.08 | 17% ± 7% | 37% ± 6% | 1.27 ± 0.08 | 10 % ± 7% | 11 % ± 6% | ns | 0.01 |
| **Tei Index (MPI)** | 0.76 ± 0.02 | -15.6% ± 4% | -6.1% ± 6.8% | 0.77 ± 0.03 | -4.0% ± 5% | 5.3% ± 5% | ns | ns |

**Abbreviations**: HR: Heart rate; Volume;d: Left ventricle interior volume during diastole; Volume;s: Left ventricle interior volume during systole; EF: Ejection Fraction;FS: Fractional shortening; CO: Cardiac output; LVAW;d: Left ventricle anterior wall diameter during diastole; LVAW;s: Left ventricle anterior wall diameter during systole; LVPW;d: Left ventricle posterior wall diameter during diastole; LVPW;s: Left ventricle posterior wall diameter during systole; LV mass (AW): Left ventricle mass (Anterior wall); LA AP: Left atrium anteroposterior diameter; LA ML: Left atrium mediolateral diameter; LA SI: Left atrium superoinferior diameter; LAV: Left atrium volume; E-peak velocity: Early-wave peak velocity; A-peak velocity: Atrial-wave peak velocity; E-A peak velocity ratio: Early-wave/Atrial-wave peak velocity ratio; IVCT: Isovolumic contraction time; IVRT: Isovolumic relaxation time; IVRT/RR: Isovolumic relaxation time/RR-Interval ratio; MPI: Myocardial performance index; LVM/BSA: Left ventricle mass/body surface area, ns: not statistically significant.

**Supplemental Table 4b. The effect of DCSL1 on male hearts.**

|  | **Controls** (n=8) | | | **DCSL1** (n=7) | | |  | |
| --- | --- | --- | --- | --- | --- | --- | --- | --- |
|  | **Baseline** | **% of change** | | **Baseline** | **% of change** | | **T-Test** | |
|  |  | **4 weeks** | **12 weeks** |  | **4 weeks** | **12 weeks** | **4 weeks** | **12 weeks** |
| **HR (bpm)** | 421 ± 15 | 3.5% ± 4% | 11% ± 6% | 467 ± 18 | 6.4% ± 4% | -1.7% ± 5% | ns | ns |
| **Volume;d (μl)** | 81.0 ± 5.6 | 10.4% ± 4% | 10.2% ± 6% | 75.1 ± 3.9 | 22.9% ± 8% | 1.1% ± 4% | ns | ns |
| **Volume;s (μl)** | 38.6 ± 4.7 | 16.3% ± 8% | 19.1% ± 14% | 36.4 ± 2.4 | 40.4% ± 15% | 10.8% ± 12% | ns | ns |
| **Stroke volume (μl)** | 42.7 ± 2.2 | 10.6% ± 8% | 6.7% ± 6% | 38.7 ± 2.2 | 10.9% ± 8% | -5.6% ± 4% | ns | ns |
| **EF (%)** | 53.8 ± 2.7 | -0.2% ± 5% | -0.3% ± 7% | 51.6 ± 1.8 | -9% ± 4% | -4.7% ± 7% | ns | ns |
| **FS (%)** | 27.8 ± 1.7 | 0% ± 7% | 0% ± 9% | 26.2 ± 1.1 | -10.1% ± 5% | -5.1% ± 8% | ns | ns |
| **CO (ml/min)** | 17.9 ± 1.2 | 14.3% ± 9% | 18.8% ± 8% | 17.9 ± 1 | 16.8% ± 8% | -8.4% ± 2% | ns | 0.01642 |
| **LVAW;d (mm)** | 1.03 ± 0.1 | 0.1% ± 7% | -1.2% ± 7% | 1.07 ± 0.03 | -20.4% ± 8% | -5.5% ± 4% | ns | ns |
| **LVAW;s (mm)** | 1.42 ± 0.1 | 0% ± 5% | 1.8% ± 6% | 1.46 ± 0.04 | -18.5% ± 7% | -3.9% ± 4% | ns | ns |
| **LVPW;d (mm)** | 1.05 ± 0.1 | 25.8% ± 11% | 28.6% ± 9% | 1.07 ± 0.1 | 13% ± 8% | 4.6% ± 4% | ns | ns |
| **LVPW;s (mm)** | 1.40 ± 0.05 | 17.6% ± 8% | 10.6% ± 5% | 1.31 ± 0.1 | 9.7% ± 7% | 1.4% ± 4% | ns | ns |
| **LV Mass (AW) Corrected** | 149 ± 8.4 | 24.6% ± 11% | 26.0% ± 11% | 147 ± 7.3 | 4.6% ± 6% | -1.1% ± 3% | ns | ns |
| **LV Mass (Teichholz)**[**^1^**](#_ENREF_1) | 88.3 ± 5 | 26% ± 18% | 41.8% ± 12% | 89.2 ± 7.1 | 23.7% ± 11% | 5.8% ± 5% | ns | 0.03324 |
| **LA AP (mm)** | 2.50 ± 0.1 | 0% ± 3% | 10.6% ± 3% | 2.41 ± 0.1 | 1.6% ± 3% | 10% ± 4% | ns | ns |
| **LA ML (mm)** | 2.78 ± 0.1 | 6.6% ± 4% | 6.9% ± 1% | 2.67 ± 0.1 | 11% ± 5% | 8.8% ± 2% | ns | ns |
| **LA SI (mm)** | 6.05 ± 0.1 | 0.8% ± 1% | 5.3% ± 1% | 5.86 ± 0.1 | 2.5% ± 2% | 4.7% ± 0% | ns | ns |
| **LA V (mm^3^)** | 22.6 ± 1.4 | 8.2% ± 7% | 22.7% ± 6% | 20.6 ± 1.5 | 16.7% ± 8% | 20.7% ± 4% | ns | ns |
| **E peak velocity (mm/s)** | 624 ± 22 | 8.7% ± 5% | 24.0% ± 8% | 625 ± 21 | 11.9% ± 5% | 25.4% ± 9% | ns | ns |
| **A peak velocity (mm/s)** | 474 ± 38 | 17.3% ± 8% | 35.3% ± 20% | 427 ± 25 | 18.6% ± 12% | 32.1% ± 12% | ns | ns |
| **E-A peak velocity ratio** | 1.41 ± 0.1 | -5.3% ± 4% | 0.1% ± 11% | 1.56 ± 0.2 | 7.6% ± 16% | -1.2% ± 9% | ns | ns |
| **IVCT (ms)** | 14.3 ± 1.4 | 5.7% ± 7% | -10.6% ± 15% | 18.1 ± 1.3 | -8.1% ± 10% | -19.9% ± 7% | ns | ns |
| **IVRT (ms)** | 18.4 ± 0.8 | -2.5% ± 6% | -8.7% ± 7% | 18.5 ± 0.5 | -0.3% ± 5% | 5% ± 7% | ns | ns |
| **IVRT/RR** | 0.13 ± 0.01 | 3.6% ± 6% | 9.8% ± 7% | 0.13 ± 0.003 | 7.8% ± 7% | 19.8% ± 10% | ns | ns |
| **Aortic peak velocity (mm/s)** | 979 ± 39 | 6.1% ± 6% | 12.3% ± 5% | 883 ± 31 | 3.7% ± 6% | 6.8% ± 6% | ns | ns |
| **LVM/BSA (mg/cm^2^)** | 1.50 ± 0.08 | 27% ± 11% | 29 % ± 11% | 1.54 ± 0.07 | 7 % ± 5% | 8% ± 2% | ns | ns |
| **Tei Index (MPI)** | 0.73 ± 0.03 | -3.9% ± 7% | -6.7% ± 6% | 0.83 ± 0.02 | -4.4% ± 5% | -6.6% ± 7% | ns | ns |

**Abbreviations**: HR: Heart rate; Volume;d: Left ventricle interior volume during diastole; Volume;s: Left ventricle interior volume during systole; EF: Ejection Fraction;FS: Fractional shortening; CO: Cardiac output; LVAW;d: Left ventricle anterior wall diameter during diastole; LVAW;s: Left ventricle anterior wall diameter during systole; LVPW;d: Left ventricle posterior wall diameter during diastole; LVPW;s: Left ventricle posterior wall diameter during systole; LV mass (AW): Left ventricle mass (Anterior wall); LA AP: Left atrium anteroposterior diameter; LA ML: Left atrium mediolateral diameter; LA SI: Left atrium superoinferior diameter; LAV: Left atrium volume; E-peak velocity: Early-wave peak velocity; A-peak velocity: Atrial-wave peak velocity; E-A peak velocity ratio: Early-wave/Atrial-wave peak velocity ratio; IVCT: Isovolumic contraction time; IVRT: Isovolumic relaxation time; IVRT/RR: Isovolumic relaxation time/RR-Interval ratio; MPI: Myocardial performance index; LVM/BSA: Left ventricle mass/body surface area; ns: not statistically significant.

**Supplemental Table 5.** The number of cells expressing dendritic cell markers is not affected by DCSL1 treatment.

| **Markers as% of CD45^+^CD11b^+^** | **Sex/Treatment** | | | | **One-way ANOVA** |
| --- | --- | --- | --- | --- | --- |
|  | Female control  N=7 | Female DCSL1  N=8 | Male control  N=8 | Male DCSL1  N=7 |  |
| CD103^+^ | 68.4 ± 2.7 | 69.2 ± 2.8 | 45.7 ± 4.3 | 43.2 ± 2.7 | P=0.0003 |
| CD11c^+^ | 89.4 ± 1.9 | 87.2 ± 1.4 | 74.5 ± 4.9 | 77.2 ± 3.0 | P=0.0051 |
| CD103^+^CD11c^+^ | 64.2 ± 2.9 | 65.7 ± 3.4 | 41.3 ± 4.0 | 41.2 ± 3.1 | P=0.0004 |

**Supplemental Table 6.** Lymphocytes are not affected by DCSL1 treatment.

|  | **Sex/Treatment** | | | | **One-way ANOVA** |
| --- | --- | --- | --- | --- | --- |
|  | Female control  N=8 | Female DCSL1  N=8 | Male control  N=6 | Male DCSL1  N=7 |  |
| **% of live cells** |  | | | | |
| CD3^+^ | 1.6 ± 0.2 | 1.9 ± 0.3 | 5.2 ± 0.6 | 3.2 ± 0.5 | P=0.0008 |
| CD56^+^ | 1.4 ± 0.3 | 1.6 ± 0.3 | 2.8 ± 0.7 | 3.6 ± 0.6 | P=0.0099 |
| **% of CD3^+^** |  | | | | |
| CD4^+^ | 14.8 ± 3.4 | 16.0 ± 2.6 | 5.4 ± 0.7 | 9.1 ± 1.2 | P=0.0042 |
| CD8a^+^ | 43.5 ± 2.2 | 49.8 ± 4.8 | 64.8 ± 2.2 | 66.4 ± 3.0 | P=0.0018 |
| CD4^+^/CD8a^+^ ratio | 0.34 | 0.32 | 0.08 | 0.13 |  |
| **% of CD3^+^CD8a^+^** |  | | | | |
| CXCR3^+^ | 0.3 ± 0.1 | 0.5 ± 0.1 | 33.1 ± 12.0 | 32.9 ± 5.7 | P=0.0001 |

**Supplemental Figure Legend**

**Supplemental Figure 1.** Flow cytometry analysis of blood CD45+ cells. Statistical analysis was performed using a two-way ANOVA with Kruskal-Wallis correction. Results are presented as mean ± SEM, N=6-8.

**Supplemental Figure 2.** Monocyte maturation to M2 macrophages was decreased by DCSL1. The 100 % line denotes a control from the same culture without MCP-1 (migration and maturation without any chemoattractant). Statistical analysis was performed using a two-way ANOVA with Kruskal-Wallis correction. Results are presented as mean ± SEM, N=3.

**Supplemental Figure 3.** Immunofluorescence staining of **A**, CD45^+^ cells in the female and male heart sections; **B**, CD45^+^TNF^+^ double positive cells in the control female and DCSL1 treated female hearts; and **C**, CD301^+^CD206^+^ double positive cells in the control female and DCSL1 treated female hearts. Arrows point to the double positive cells. Scale bar = 20 μm. Biological repeats n=4.

**Supplemental Figure 4.** Differences in cardiac collagen deposition. **A,** Representative images showing differences in collagen deposition in a female and male heart sections stained with picrosirius red. Scale bar = 100 μm. **B**, Higher magnification images (upper panel) under polarized light. Scale bar = 20 μm.The area stained by picrosirius red corresponding to collagen deposition was calculated using Image J software (lower left panel). Green, yellow, orange and red corresponding to fibril maturation where green corresponds to the thinnest and least mature and red the most mature. Changes in color were used to calculate the proportion of each fiber type in the hearts (lower right panel) with respect to the total area. **C**, Representative images of collagen deposition in control versus DCSL1-treated female heart. Sections were stained with picrosirius red. Scale bar = 100 μm. **D**, Images (upper panel) of picrosirius red stained heart sections under polarized light. Scale bar = 20 μm. The lower left panel shows a computed area stained by picrosirius red per mm^2^. The lower right panel shows the calculated proportion of each fiber type in the hearts (as in B) with respect to the total area. Biological repeats n=3, at least 3 sections and 3 areas per each heart section were
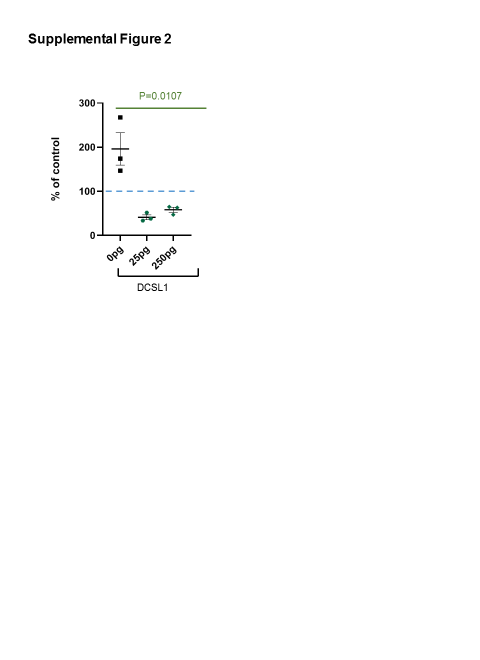

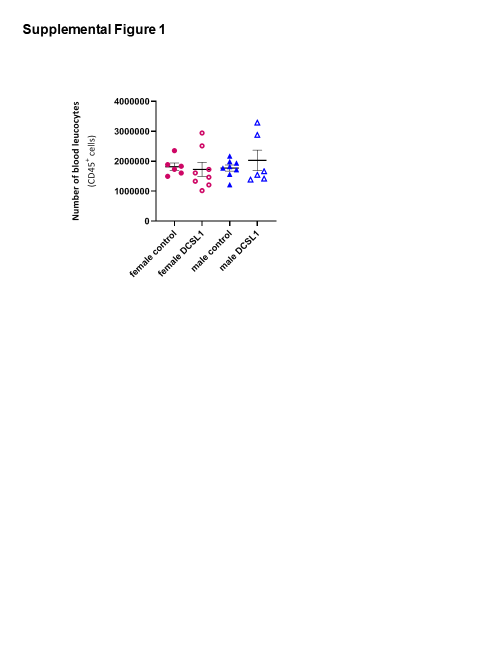
analyzed. Statistical analysis was performed using Student’s t-test for B and D.


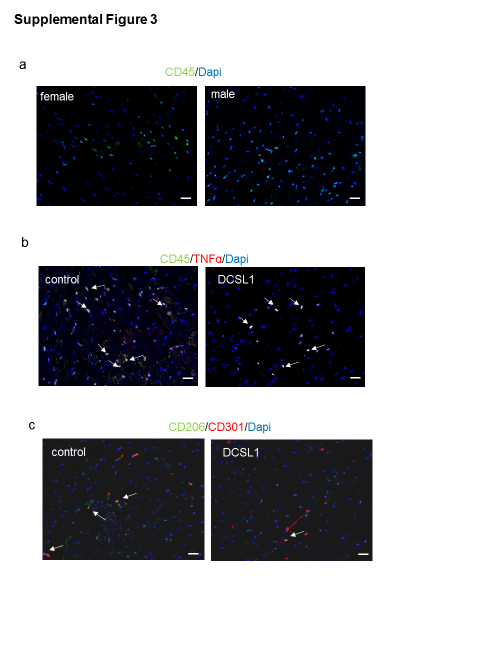


**
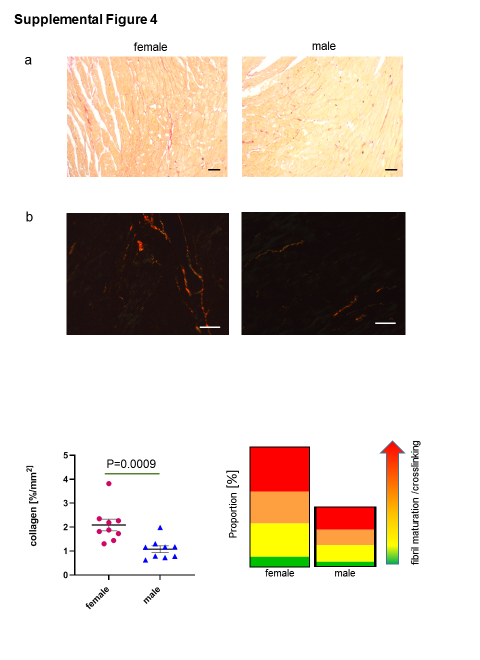

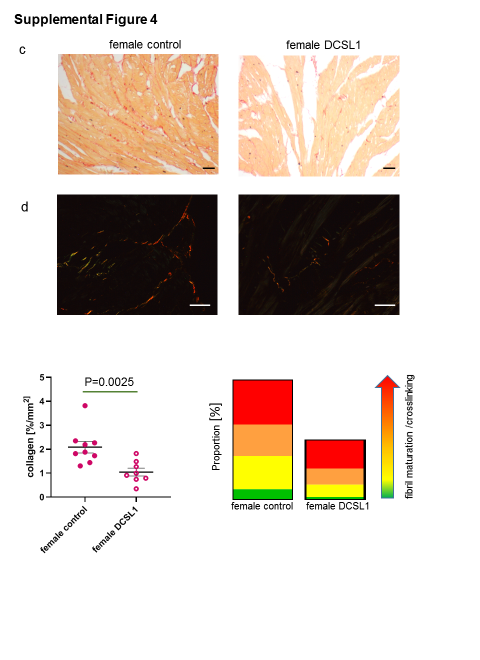
Supplemental Figure** **4**
